# Lipid lowering and Alzheimer disease risk: A mendelian randomization study

**DOI:** 10.1002/ana.25642

**Published:** 2019-12-13

**Authors:** Dylan M. Williams, Chris Finan, Amand F. Schmidt, Stephen Burgess, Aroon D. Hingorani

**Affiliations:** ^1^ Medical Research Council Unit for Lifelong Health and Ageing at University College London University College London London United Kingdom; ^2^ Department of Medical Epidemiology & Biostatistics Karolinska Institute Stockholm Sweden; ^3^ Institute of Cardiovascular Science, Faculty of Population Health University College London London United Kingdom; ^4^ Health Data Research UK London United Kingdom; ^5^ British Heart Foundation University College London Research Accelerator London United Kingdom; ^6^ Department of Cardiology, Division Heart and Lungs University Medical Center Utrecht Utrecht the Netherlands; ^7^ Medical Research Council Biostatistics Unit University of Cambridge Cambridge United Kingdom; ^8^ Cardiovascular Epidemiology Unit, Department of Public Health and Primary Care University of Cambridge Cambridge United Kingdom

## Abstract

**Objective:**

To examine whether genetic variation affecting the expression or function of lipid‐lowering drug targets is associated with Alzheimer disease (AD) risk, to evaluate the potential impact of long‐term exposure to corresponding therapeutics.

**Methods:**

We conducted Mendelian randomization analyses using variants in genes that encode the protein targets of several approved lipid‐lowering drug classes: *HMGCR* (encoding the target for statins), *PCSK9* (encoding the target for PCSK9 inhibitors, eg, evolocumab and alirocumab), *NPC1L1* (encoding the target for ezetimibe), and *APOB* (encoding the target of mipomersen). Variants were weighted by associations with low‐density lipoprotein cholesterol (LDL‐C) using data from lipid genetics consortia (n up to 295,826). We meta‐analyzed Mendelian randomization estimates for regional variants weighted by LDL‐C on AD risk from 2 large samples (total n = 24,718 cases, 56,685 controls).

**Results:**

Models for *HMGCR, APOB*, and *NPC1L1* did not suggest that the use of related lipid‐lowering drug classes would affect AD risk. In contrast, genetically instrumented exposure to PCSK9 inhibitors was predicted to increase AD risk in both of the AD samples (combined odds ratio per standard deviation lower LDL‐C inducible by the drug target = 1.45, 95% confidence interval = 1.23–1.69). This risk increase was opposite to, although more modest than, the degree of protection from coronary artery disease predicted by these same methods for PCSK9 inhibition.

**Interpretation:**

We did not identify genetic support for the repurposing of statins, ezetimibe, or mipomersen for AD prevention. Notwithstanding caveats to this genetic evidence, pharmacovigilance for AD risk among users of PCSK9 inhibitors may be warranted. ANN NEUROL 2020;87:30–39

There are no preventive or disease‐modifying treatments for Alzheimer disease (AD). Expanding the indications of drugs of proven efficacy into other indications might be an effective strategy to provide new clinical treatments and preventative medicines for AD.^1^ Opportunities for indication expansion should be widespread, considering arguments based on first principles,^2^ and empirical evidence from genome‐wide association studies (GWASs) showing that the same gene can influence risk of more than one disease (pleiotropy).^3^

Medications that decrease circulating low‐density lipoprotein cholesterol (LDL‐C), such as statins, have been proposed as candidate therapies for AD. Hyperlipidemia in midlife is a risk factor for late onset AD in prospective epidemiological studies,^4^ and associations of higher LDL‐C with increased cerebral β‐amyloid load have also been observed in autopsy and in vivo imaging studies.^5^, ^6^ Similarly, AD risk is lower among statin users, and this association appears to be more pronounced with longer treatment exposure and the use of more potent drugs.^7^ In contrast, corresponding observational data on other lipid‐lowering drug classes are scant and inconclusive.^7^ Large randomized controlled trials (RCTs) may help to clarify the effects of dyslipidemia treatments on AD incidence without confounding, but such evidence is limited,^8^ and the slowly evolving pathogenesis of AD (over at least 1 decade)^9^, ^10^ means it is ill‐suited as an endpoint in trials of lipid‐lowering drugs with relatively short periods of intervention and follow‐up (typically 2–5 years).

Genetic epidemiology provides another means to address these questions. The expression or function of protein drug targets can be influenced by variants within or near the genes that encode them, and the genetic effects can be used to anticipate the effects of drug action.^11^ Because genotypes are inherited randomly at conception in an analogous manner to treatment allocation in clinical trials, associations of variants with biomarkers and disease outcomes are not expected to be subject to biases from confounding and reverse causation seen in other types of observational epidemiology—a principle leveraged in an approach known as “Mendelian randomization” (MR).^12^ Moreover, genotypes are mostly anticipated to confer lifelong differences in traits. Hence, MR studies can help to guide drug target validation by predicting the consequences of long‐term therapeutic exposure.^13^ In this study, we examined whether AD risk is influenced by variation in the genes encoding the targets of a range of medications that are currently licensed and recommended for the treatment of primary or familial hypercholesterolemia to prevent coronary heart disease.

## Subjects and Methods

### *Study Design*

We used a 2‐sample MR study design for these analyses,^14^ based mainly on genetic variants located in or near genes that encode the relevant drug targets (*cis*‐MR).^13^ We first identified differences in circulating LDL‐C that are associated with single nucleotide polymorphisms (SNPs) at the genomic regions of interest in sets of publicly available genetic association summary statistics, because the lowering of LDL‐C in circulation is a major, established physiological response produced by the use of the lipid‐lowering therapeutics. We then combined the LDL‐C association statistics for variant sets with corresponding genome‐wide association statistics for AD risk from 2 large, independent case–control datasets, to predict the effects of drug use on AD development.

**Figure 1:**
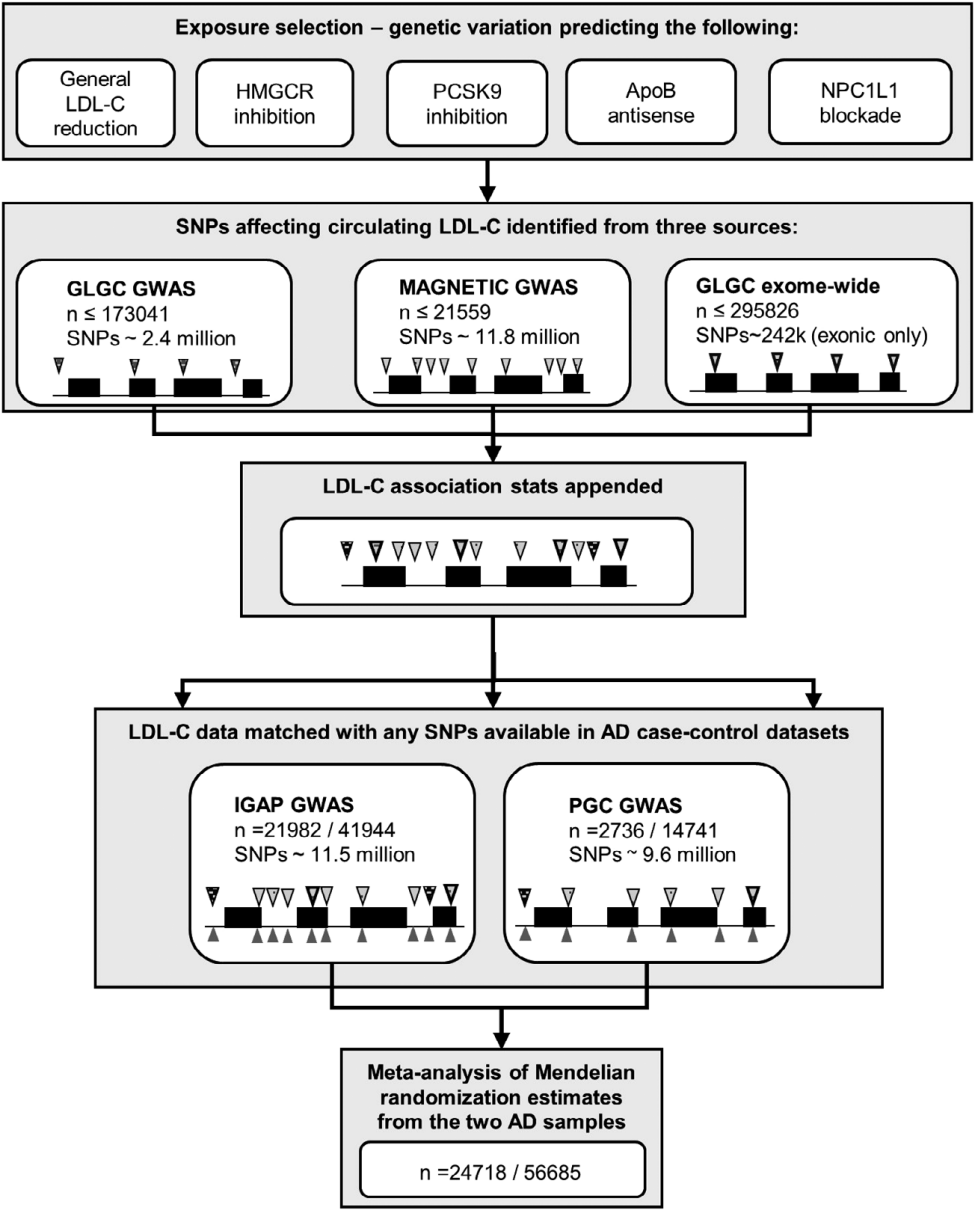
Study overview. Black lines depict an illustrative gene region, with raised boxes showing exons. Wedges represent the presence of specific genetic variants (single nucleotide polymorphisms [SNPs]) measured throughout the gene. Different patterns of the wedges illustrate the combination of sources of low‐density lipoprotein cholesterol (LDL‐C) association statistics across variants from 1 of 3 LDL‐C genome‐wide association studies (GWASs; second and third panels). The pairing of wedges above and below the gene outline in panel 4 depicts the harmonization of appended LDL‐C statistics with corresponding estimates of Alzheimer disease (AD) risk for each variant within the 2 AD datasets separately. The varying number of wedges present across each dataset represents the differences in densities of SNPs that were genotyped or imputed in the data. GLGC = Global Lipid Genetics Consortium; IGAP = International Genomics of Alzheimer's Project; PGC = Psychiatric Genomics Consortium.

As depicted in the study overview (Fig 1), we performed 5 main MR analyses: one assessing the effect of a general reduction of LDL‐C on AD risk (ie, achievable by any means), and the remainder for specific effects of variation at 4 gene regions (*HMGCR, PCSK9, APOB*, and *NPC1L1*) that are indicative of the long‐term use of different lipid‐lowering drug classes that are currently licensed and guideline‐supported for lowering LDL‐C in the primary prevention of coronary heart disease, familial hypercholesterolemia, or both. These genes encode the targets of statins (HMG‐CoA reductase [HMGCR]), proprotein convertase subtilisin/kexin type 9 (PCSK9) inhibitors (eg, evolocumab and alirocumab), apolipoprotein B‐100 (ApoB) antisense classes (eg, mipomersen), and NPC1‐like intracellular cholesterol transporter 1 (NPC1L1) inhibitors (eg, ezetimibe), respectively.

In post hoc analyses to follow up on genetic analyses modeling PCSK9 inhibition in relation to AD risk that were weighted by LDL‐C, we also examined whether genetically indexed variation in circulating PCSK9 concentration is associated with AD risk.

### *Deriving SNP Associations with LDL‐C*

SNP–LDL‐C association statistics were extracted from 3 GWASs of circulating LDL‐C concentrations—2 from the Global Lipid Genetics Consortium^15^, ^16^ and 1 from the MAGNETIC consortium.^17^ These contained different sets of genotyped and imputed SNP data and varying sample sizes, and hence estimated any genetic effect with varying precision. Due to partial overlap between the GWAS samples, the 3 sets of estimates were appended together, rather than combined in a meta‐analysis to prevent double counting (see Fig 1). Where the same SNP–LDL‐C associations were present in more than one GWAS dataset, we retained the estimate from the largest of the samples. In combining these sets of GWAS summary statistics, we aimed to leverage the maximum degree of genetic variation measured at each region (optimizing statistical power to detect associations of regional variation with AD risk). The sample sizes for SNP–LDL‐C association statistics ranged from 14,004 to 295,826. Each of the LDL‐C GWASs had homogeneous samples (all of European ancestry) and was modeled with consistent statistical procedures and transformations of the LDL‐C distributions.

For general LDL‐C models, we used independent SNPs throughout the genome that had replicated evidence for association with LDL‐C in any of the 3 GWAS datasets. We excluded a SNP in *APOE* on chromosome 19 due to a strong, established pleiotropic effect of this locus on AD risk, and another variant on chromosome 19 that exhibited linkage disequilibrium (LD) with the SNPs that constitute the ε2/3/4 genotypes in *APOE*. A total of 59 SNPs remained after LD clumping (steps described further in the Statistical Analysis section) and were taken forward to harmonize with AD data. In primary gene region models, we extracted data on all SNPs within 1 kilobase in either direction of the start and stop coordinates for the genes, according to Human Genome reference release GRCh37 positions. In secondary models for gene regions, we expanded the selection to all SNPs within ±100 kilobases of the genes’ coordinates. Data on the beta coefficients, standard errors, coded effect, and alternate alleles and allele frequencies per SNP were extracted to combine with summary statistics from the AD datasets.

Analyses to assess the effect of modifying circulating PCSK9 on AD risk were based on SNP–PCSK9 association statistics for 6 variants identified in a GWAS of circulating PCSK9 in suspected or confirmed coronary artery disease (CAD) patients of European ancestry.^18^ We used SNP–PCSK9 association statistics from a subgroup analysis of statin‐naive participants in the GWAS (n = 2,022).

### *AD Data*

We assessed whether LDL‐associated variants also associate with late onset AD risk (age of onset ≥65 years) using data from 2 large genome‐wide analyses from the International Genomics of Alzheimer's Project (IGAP) and the Psychiatric Genomics Consortium (PGC).^19^, ^20^ The total sample size was 24,718 cases and 56,685 controls, all of whom had European ancestry (summary details are given in Table 1). Full information on genetic quality control procedures and association modeling are provided in the study references.^19^, ^20^

**Table 1:** Summary Information on AD Datasets

	Study	
	IGAP^19^	PGC^20^
Cases, n	21,982	2,736
Controls, n	41,944	14,741
Sample sources	Case–control studies, longitudinal cohorts	Register‐based follow‐up of twin cohorts/case–control DemGene study
Country of origin	Europe, Canada, USA	Sweden/Norway
Case mean AAO, yr^a^	71.1–82.6	77.0–80.5
Control mean AAE, yr^a^	51.0–78.9	58.8–75.5
% female, cases/controls	59.3–67.3/51.8–60.6	52.5–66.4/48.0–51.1
AD ascertainment	Clinical assessment, MRI‐ or autopsy‐confirmed, and/or diagnoses from health care records	Clinical assessment and/or diagnoses from health care records
Genetic data	Genome‐wide genotyping, imputation using 1000 Genomes project, phase 2 release	Genome‐wide genotyping, imputation using 1000 Genomes project, phase 3 release

a Ranges of mean or percentage values are presented for IGAP stage 1 and PGC data, which combined 4 consortia/studies and 3 samples in these genome‐wide association studies, respectively.

AAE = age at examination or last follow‐up; AAO = age at onset; AD = Alzheimer disease; IGAP = International Genomics of Alzheimer's Project; MRI = magnetic resonance imaging; PGC = Psychiatric Genomics Consortium.

### *Positive Control Analyses*

To help validate the chosen modeling strategy, we also merged the same sets of LDL‐C summary statistics with genetic data on the risk of CAD and type 2 diabetes mellitus (T2D), for which we may expect to see effects of LDL‐C modification (ie, positive control analyses). These models used summary GWAS data on CAD risk from the CARDIoGRAM consortium (n = 22,233 cases, 64,762 controls)^21^, ^22^ and T2D risk from the DIAGRAM consortium (n = 12,171 cases, 56,862 controls).^22^ Samples included participants of European ancestry only.

### *Statistical Analysis*

Data for SNP associations with LDL‐C or PCSK9 and with risk of AD or cardiometabolic outcomes were harmonized to match coded effect alleles consistently; we excluded ambiguous SNPs from analyses (those with palindromic genotypes and minor allele frequencies between 0.4 and 0.5).

To assess a potential effect of LDL‐C lowering on AD, regardless of drug target, we used the fixed‐effects inverse variance weighted (IVW) meta‐analysis method, which combines ratios of coefficients (Wald estimators) for the association estimates of each eligible SNP with LDL‐C and AD.^23^ We repeated analyses using alternative methods—weighted median and MR Egger estimators—which provide different degrees of robustness to bias from genetic pleiotropy.^24^, ^25^

For the primary gene (drug target)‐specific models, data on all successfully harmonized SNPs within the gene regions (±1 kilobase flank) were eligible for inclusion. We then used LD clumping to remove the excess of most highly correlated variants at each locus (with pairwise *r* ^2^ values >0.6), retaining the member of each correlated pair with the lowest *p* value for association with LDL‐C. Remaining SNPs were modeled together using a principal components (PC)‐based approach to handle estimates from correlated variants.^26^ This method relies on the use of reference data to estimate the correlations between variants in summary GWAS datasets, for which we used correlation matrices derived from 503 participants of European ancestry in the 1000 Genomes project, phase 3.^27^ We tested the robustness of using these reference data by inputting several matrix types from different subpopulations of the 1000 Genomes project sample, including the actual correlations from the PGC data. We also tested the consistency of PC estimates by specifying 3 values of κ (0.9, 0.99, and 0.999), which would be expected to model between 90 and 99.9% of variance in LDL‐C attributable to genetic variants in each gene region (results from the 99% models are presented in each figure).^26^ For comparison, we also conducted analyses for gene region models with a wider range of SNPs for inclusion (within ±100 kilobases of the genes), and with MR estimates provided by the IVW method to enroll only independent SNPs with GWAS significant associations with LDL‐C (SNP–LDL association *p* values <5 × 10^−8^). Independence between SNPs was defined by pairwise *r* ^*2*^ values <0.2 in one set of models, and then repeated with SNPs clumped using a more conservative threshold (*r* ^2^ < 0.01). Funnel plots were produced to test whether overall MR estimates were consistent with the estimates provided by the most precise individual SNP estimators, and leave‐one‐out plots were produced to examine whether models were influenced by any individual outlying SNP estimates.^28^ Using LD‐clumped variants, we also conducted MR Egger and weighted median models for any gene region–AD variant set that included 3 or more SNPs. We also conducted both PC and LD‐clumped gene region models for analyses of cardiometabolic outcomes.

For both general LDL‐C and gene (drug target)‐specific models, we opted to analyze effects on AD risk separately in the 2 AD GWAS datasets, due to these having varying SNP data available and different sample structures (a mix of matched case–control studies, samples from longitudinal cohorts, and health care register–derived samples). Finally, we combined the 2 results for each trait–AD association into overall MR estimates using fixed‐effects IVW meta‐analyses. Results were scaled to express odds ratios (ORs) for AD and cardiometabolic outcomes per 1 standard deviation (SD) lower LDL‐C, to indicate the expected directions of effect that would follow from the use of lipid‐lowering drugs.

Post hoc analyses for circulating PCSK9 involved the same steps and MR methods used to model the effect of a general LDL‐C lowering on AD risk. Again, we also estimated the effects of lowering PCSK9 on CAD for comparison. Because SNP–PCSK9 associations were expressed in log_n_‐transformed PCSK9 units, results from these MR models were scaled to show CAD and AD risk differences per halving of circulating PCSK9.

Analyses were undertaken in Stata version 15 (StataCorp, College Station, TX), PLINK 1.9 (http://www.cog-genomics.org/plink/1.9/), and R version 3.4.1, with the aid of the packages MR Base and MendelianRandomization.^29^, ^30^

This research involved the reuse of existing anonymized, study‐level summary data only. All of the original studies had received informed consent from participants and ethical approvals.

## Results

After harmonizing exposure (LDL‐C or PCSK9) and disease outcome genetic associations, and subsequent LD clumping, main MR models included between 2 and 55 variants (Supplementary Tables 1–5).

**Figure 2:**
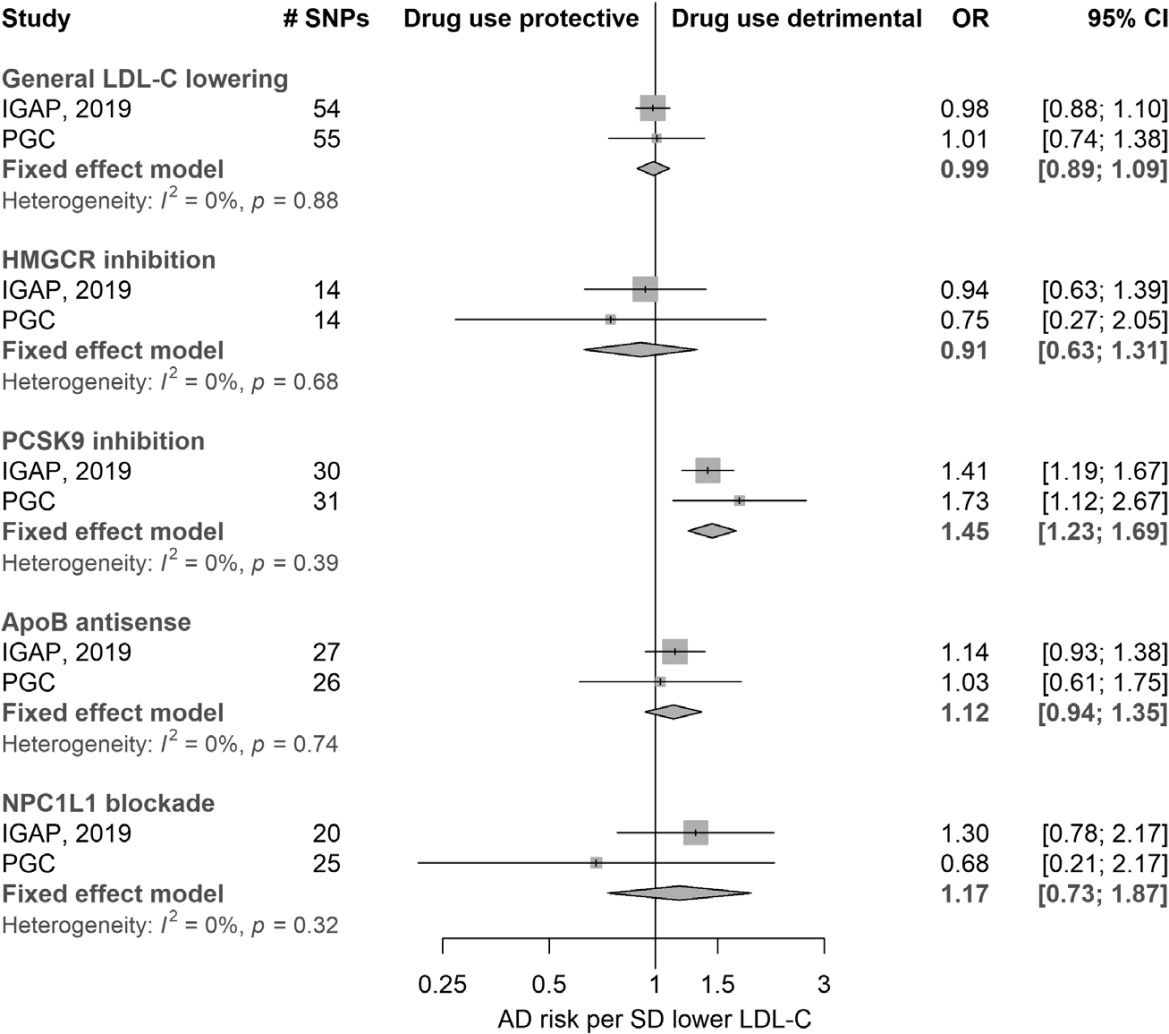
Meta‐analysis of Mendelian randomization estimates for Alzheimer disease (AD) risk according to a lifelong reduction in circulating low‐density lipoprotein cholesterol (LDL‐C) and exposure to the modulation of several related drug targets (n = 24,718 cases, 56,685 controls). The first group of results shows estimates for the effect of a general, long‐term reduction of LDL‐C (achievable by any means) on AD risk. The second to fifth group labels are representative of genetic variation at gene regions (*HMGCR*, *PCSK9, APOB*, and *NPC1L1*) that predict the effects of specific therapeutic target modulation, followed by example drug classes that affect these targets. CI = confidence interval; IGAP = International Genomics of Alzheimer's Project; OR = odds ratio; PGC = Psychiatric Genomics Consortium; SD = standard deviation; SNP = single nucleotide polymorphism.

A general, long‐term reduction in circulating LDL‐C, indexed using eligible variants throughout the genome, was not estimated to affect AD risk (Fig 2). Findings were also null in alternate MR analyses using weighted median and MR Egger methods (Table 2), and these findings did not suggest any notable bias from directional pleiotropy or weak instruments in these models; for example, in analyses of the IGAP dataset, the MR Egger intercept test was 1.00 (*p* = 0.39), with I^2^_gx_ of 98.7% in both datasets.

**Table 2:** MR Estimates for the Effect of LDL‐C Lowering on AD Risk in IGAP and PGC Datasets

Study	Method	Odds Ratio	95% CI	*p*
IGAP	IVW	0.98	0.88–1.10	0.75
	MR Egger	1.04	0.88–1.22	0.67
	MR Egger intercept test	1.00	0.99–1.00	0.39
	Wtd median	0.95	0.82–1.11	0.53
PGC	IVW	1.01	0.74–1.38	0.96
	MR Egger	1.41	0.89–2.25	0.15
	MR Egger intercept test	0.98	0.96–1.00	0.06
	Wtd median	1.19	0.81–1.75	0.38

IVW results are illustrated in the top section of Figure 2. Odds ratios and 95% CIs are per 1 standard deviation lower LDL‐C. IGAP and PGC models included 54 and 55 single nucleotide polymorphisms, respectively.

AD = Alzheimer disease; CI = confidence interval; IGAP = International Genomics of Alzheimer's Project; IVW = inverse variance weighted; LDL‐C = low‐density lipoprotein cholesterol; MR = Mendelian randomization; PGC = Psychiatric Genomics Consortium; Wtd = weighted.

Gene region models using the PC‐based approach for *HMGCR, APOB*, and *NPC1L1* did not provide clear evidence to suggest that the use of the corresponding lipid‐lowering drug classes would affect AD risk (see Fig 2). Results for these regions were largely consistent when repeated with IVW models; all point estimates for *HMGCR* were on the side of neuroprotection and all estimates for *APOB* and *NPC1L1* were on the side of risk, but confidence intervals (CIs) in all instances were wide and included the null. In contrast, variants in the vicinity of *PCSK9* implied that exposure to PCSK9 inhibitors could increase the risk of AD (OR = 1.45, 95% CI = 1.23–1.69, *p* = 4.4 × 10^−6^). Estimates were similar in meta‐analyses of IVW models with liberal clumping (OR = 1.37, 95% CI = 1.12–1.66) and conservative clumping (OR = 1.44, 95% CI = 0.94–2.20). There was no evidence of heterogeneity between estimates from the 2 AD samples (*p* values for heterogeneity tests in all models ≥0.11). Funnel plots did not indicate asymmetry in SNP estimates across the scale of precision in IVW models, and leave‐one‐out plots did not suggest that any of the IVW findings were greatly influenced by single outlying Wald estimators (plots available on request). MR Egger and weighted median results for LD‐clumped variant sets are shown in Supplementary Table 6. In general, these did not deviate greatly from IVW findings, but most estimates were very imprecise (particularly those from MR Egger). Point estimates varied widely between IVW, weighted median, and MR Egger methods for PCSK9 inhibition and AD risk in IGAP data, but were more consistent in the PGC dataset.

Sensitivity analyses indicated that the choice of reference data used to model variant correlations would not be expected to influence findings from PC models notably. ORs for the effects of PCSK9 inhibition on AD risk in the PGC sample ranged from 1.64 (95% CI = 1.17–2.31) per SD lower LDL‐C using allele frequencies from a sample with Finnish ancestry to 1.73 (95% CI = 1.12–2.66) using allele frequencies of samples with ancestries from across Europe. In comparison, the corresponding estimate using the sample's actual allele frequencies was 1.69 (95% CI = 1.14–2.52). Changing input parameters for PC models introduced some slight variation in the magnitudes of estimates. For example, using κ = 0.90 or κ = 0.999 instead of 0.99 in PC models (ie, enrolling either 90% or 99.9% of variance in LDL‐C concentrations attributable to genetic variation in the gene region) yielded meta‐analysis effect estimates for PCSK9 inhibition on AD risk of 1.35 (95% CI = 1.09–1.67) and 1.62 (95% CI = 1.41–1.86), respectively. Despite these varying magnitudes of estimates, the use of different PC model permutations would not change the overall interpretation of these findings.

**Figure 3:**
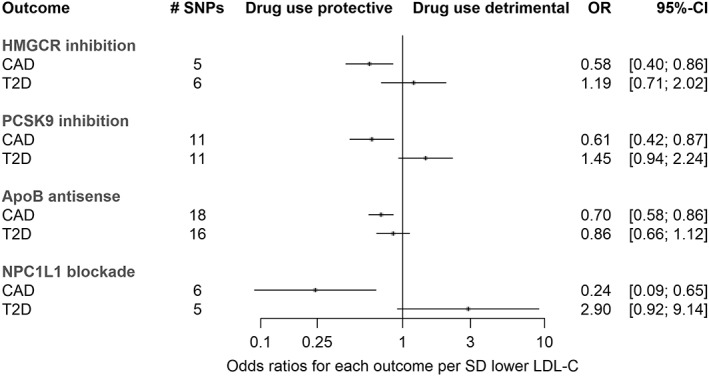
Mendelian randomization estimates for the effects of exposure to drug target modulation on coronary artery disease (CAD) and type 2 diabetes mellitus (T2D). Results were produced using variants within gene regions (±1 kilobase flanks) and the principal components–based method, as per analyses for the Alzheimer disease results presented in Figure 1. Sample sizes: CAD, n = 22,233 cases, 64,762 controls; T2D, n = 12,171 cases, 56,862 controls. CI = confidence interval; LDL‐C = low‐density lipoprotein cholesterol; OR = odds ratio; SD = standard deviation; SNP = single nucleotide polymorphism.

Using the same approach to predict the effects of long‐term modulation of these targets on cardiometabolic outcomes (positive control models), genetically predicted exposures to all lipid‐lowering drug classes were associated with a lower risk of CAD (Fig 3). There were trends for increased risk of T2D with predicted exposure to statins and inhibitors of PCSK9 and NPC1L1 (as has been observed previously^31^, ^32^), although results from *NPC1L1* variants were estimated with limited precision.

Given the finding that genetic indexing of PCSK9 inhibition predicted higher AD risk in our main models, we further examined whether differences in exposure to circulating PCSK9 may be related to AD risk using MR, and compared these results to the predicted effect of circulating PCSK9 variation on CAD (Table 3). As expected, lowering PCSK9 concentration (which correlates modestly with lower circulating LDL‐C) was predicted to reduce CAD risk. In IVW models for AD, point estimates from IGAP and PGC were both on the side of risk per halving of circulating PCSK9, but the IGAP sample result and the meta‐analysis finding both included the null. Point estimates from MR Egger and weighted median methods were very similar to IVW estimates from both AD samples, but the findings had very little precision due to the use of few available SNPs to index PCSK9 differences.

**Table 3:** MR Estimates for the Effect of Lowering PCSK9 on CAD and AD Risk

Study	SNPs, n	Method	Odds Ratio	95% CI	*p*
CAD	2	IVW	0.57	0.36–0.89	0.01
IGAP	3	IVW	1.10	0.85–1.43	0.47
		MR Egger	1.10	0.67–1.81	0.78
		MR Egger intercept test	1.00	0.93–1.07	0.99
		Wtd median	1.09	0.81–1.47	0.56
PGC	3	IVW	1.98	1.02–3.81	0.04
		MR Egger	1.95	0.58–6.54	0.48
		MR Egger intercept test	1.00	0.84–1.19	0.98
		Wtd median	1.72	0.81–3.65	0.16
AD meta‐analysis	3	IVW	1.19	0.94–1.51	0.16

Odds ratios and 95% CIs are per halving of circulating PCSK9 concentrations.

AD = Alzheimer disease; CAD = coronary artery disease; CI = confidence interval; IGAP = International Genomics of Alzheimer's Project; IVW = inverse variance weighted; MR = Mendelian randomization; PGC = Psychiatric Genomics Consortium; SNP = single nucleotide polymorphism; Wtd = weighted.

## Discussion

This MR study did not identify support for the repurposing of statins, or medications that inhibit NPC1L1 or block ApoB production, to delay or prevent AD onset. Our results also raise the possibility (but in no way confirm) that exposure to PCSK9 inhibitors might predispose individuals to AD, although with the magnitude of relative risk observed being smaller than for the estimated degree of protection from CAD.

If the use of lipid‐lowering medications in general does not affect AD risk, this may signify the absence of a substantial role of primary hypercholesterolemia in AD etiology. The only major RCT to have addressed the effect of therapeutic LDL‐C reduction on dementia risk to date found equal incidence (0.3%) in both simvastatin and placebo‐allocated arms after 5 years of follow‐up (albeit in a sample aged 40–80 years at baseline, thus including many individuals too young to be at risk of late onset AD).^33^ Taking this finding alongside no prominent genetic associations observed for general LDL‐C lowering (as others have observed previously),^34^, ^35^ and unclear or null results for HMGCR and 2 other drug targets, suggests that previous prospective observational associations of hyperlipidemia with AD, and of higher LDL‐C with AD‐related neuropathology, may have been overstated due to residual confounding. Associations of lipid‐lowering drug use with AD risk would also be prone to bias via confounding by indication.^36^

A previous analysis has also addressed whether variation at *HMGCR* and *PCSK9* is associated with AD risk using MR.^37^ After excluding pleiotropic variation at *APOE*, the authors found no association of a genetically predicted reduction in LDL‐C exposure with AD in a sample that overlaps with one of those in the current analyses (a previous IGAP GWAS from 2013).^38^ Their result using *HMGCR* and *PCSK9* variation (combined in a single IVW model) from IGAP data had a consistent direction and similar magnitude of association as in our findings, but conflated the potentially independent associations of the different gene regions with AD, and may have lacked precision due to the use of fewer variants and a smaller sample.^37^ Using a more comprehensive survey of genetic variation at *PCSK9* separately in 2 large AD datasets, our analyses yielded consistent estimates of higher AD risk from lower PCSK9 function, regardless of the MR method used. We also report tentative MR evidence to suggest that lower circulating PCSK9 might reduce AD risk, although these findings were inconclusive, because the precision of the models was limited by the use of few variants with which to proxy exposure to PCSK9 concentrations.

Considering the full range of our findings together, if PCSK9 function has a role in determining AD risk, this is likely to be through pathways other than the regulation of peripheral LDL‐C. Experimental evidence has linked PCSK9 dysfunction to cerebral β‐amyloid production and neuronal cell death—both hallmarks of AD.^39^ PCSK9 may also promote neurogenesis during development,^40^ so any impact on AD risk could potentially be mediated by establishing “brain reserve” in early life,^41^ as opposed to a hastening of AD pathogenesis in adulthood. However, concerns of adverse neurocognitive effects of PCSK9 inhibition have been raised from a network meta‐analysis of RCTs of PCSK9 inhibitors in middle‐aged and older individuals.^42^ Another MR analysis has also suggested potentially detrimental effects of lower PCSK9 function on cognitive ability in a cohort of participants aged 38 to 73 years.^43^ A further consideration is whether PCSK9 inhibition might harm the cerebral vasculature (independently of effects on atherosclerosis). Cerebrovascular damage might influence AD neurodegeneration directly,^44^ or affect risk estimates if AD case samples include misclassified vascular (or mixed) dementia patients. However, other MR studies and RCTs have found conflicting evidence of the relationship of PCSK9 inhibition with stroke risk,^26^, ^45^, ^46^ and we have no knowledge of evidence implicating PCSK9 function in cerebral small vessel disease or vascular dementia.

Given that PCSK9 inhibitors are an efficacious second‐line drug class for the treatment of primary hypercholesterolemia and mixed dyslipidemia, we present these suggestive findings cautiously. We note that the point estimates from the much larger sample (IGAP) were less pronounced than those from the smaller sample across the various types of models, although several estimates from IGAP were still of a concerning magnitude individually. Moreover, when considering SNPs with the greatest evidence of functional relevance in the *PCSK9* region, such as the rare missense variant rs11591147, genotype–AD associations were on the side of higher risk per LDL‐lowering allele but were not statistically significant individually in either dataset or when meta‐analyzed. Further genetic studies can help to confirm or refute these results in several ways, including (1) repeating MR models of PCSK9 function in larger GWASs of AD risk, which would ideally include direct genotyping of functionally relevant SNPs in the *PCSK9* gene region; (2) assessing the associations of *PCSK9* variants with AD endophenotypes, for example, from large‐scale GWASs of fluid and/or imaging biomarkers of β‐amyloid load; and (3) recall‐by‐genotype studies to examine the presence of subclinical AD pathologies and symptoms in older individuals carrying loss‐ or gain‐of‐function *PCSK9* mutations. Because PCSK9 inhibitors have been licensed since 2015 in several territories, pharmacoepidemiologic studies of the consequences of PCSK9 inhibitor use versus other lipid‐lowering agents for AD risk could also be conducted with large samples of individuals in national or insurance‐based prescription registers, as could the follow‐up of AD risk differences among past participants in PCSK9 inhibitor trials.

The principal strength of this study was the use of 2 large AD samples in which to test for the consistency, and improve the precision, of MR estimates. The merging of LDL‐C summary statistics and use of methodology to combine correlated variation in MR models allowed us to predict the effects of manipulating drug targets more precisely, even when using variants solely within gene regions. Using *cis‐*acting variants alone should be less prone to bias from horizontal pleiotropy than the use of variation across wide flanking regions, because there is a higher probability of SNPs incidentally tagging AD risk variants elsewhere in the genome with increasing physical distance from the gene encoding a target of interest.

Limitations include the prediction solely of on‐target effects of drug use by our models; they do not encapsulate off‐target consequences of using the related therapeutic classes. Second, genetics may not be informative about particular pharmacological aspects of drug exposure. For instance, monoclonal antibodies, such as evolocumab and alirocumab, are not expected to cross the blood–brain barrier (at least while the barrier's impermeability to large molecules is intact).^47^ Hence, peripherally administered PCSK9 inhibitors may not lead to cerebral exposure, which means the genetically‐instrumented risk might not be relevant in therapeutic practice. Analogous circumstances have been examined for T2D, where evidence from human genetics suggests reduced PCSK9 activity leads to greater diabetes risk, but there is no clear evidence to show that this risk increase has borne out among RCT participants exposed to PCSK9 inhibitors.^31^, ^48^ The disparities may be reconciled if localized PCSK9 function in pancreatic tissue were to affect glycemic control independently of any inhibition of PCSK9 in circulation, as has been suggested by data from tissue‐selective *Pcsk9* knockout mice.^49^ If data from larger GWASs of circulating PCSK9 become available, improved MR analyses of circulating PCSK9 concentrations and AD risk (ie, using multiple genome‐wide SNPs to index differences in exposure) would help to evaluate the role of peripheral PCSK9 inhibition specifically. Third, given that genetic effects are often lifelong, we cannot distinguish critical periods of exposure to these targets in which disease risk might be specifically affected, such as neurodevelopment. Fourth, differences between the IGAP and PGC samples are also worth noting; in particular, a modest proportion of AD cases in the PGC sample were diagnosed from linkage to in‐ and outpatient health care records after the latest clinical assessments of these individuals were conducted (up to 741 cases, 27% of the PGC case sample). Individuals with other forms of dementia may have been misclassified as AD cases, or AD cases as controls, at higher rates with this type of ascertainment than in case–control or cohort studies with direct clinical assessments and/or pathological confirmation of AD.^50^ This could bias exposure–outcome association estimates if the exposures under study relate to the chance of misclassification differentially, for example, if an exposure increases the risk of hospitalization and, therefore, raises the likelihood of AD detection. However, this would not be expected to explain an inverse association of a trait affecting CAD risk with lower AD risk, as we observe for *PCSK9* variation. Furthermore, a large majority of the cases in the PGC sample, as well as IGAP, were diagnosed reliably with AD at memory clinics according to standard criteria.

### *Conclusions*

Evidence from this study does not support RCTs to investigate the repurposing of statins, or inhibitors of NPC1L1‐ or ApoB‐blocking therapeutics, for AD prevention. Notwithstanding the several caveats discussed here, pharmacovigilance for neurocognitive effects and the scope for increased AD burden in users of PCSK9 inhibitors may be warranted.

## Author Contributions

All authors contributed to the conception and design of the study; D.M.W. conducted the analysis of data, and contributed to drafting the text and preparing the figures.

## Potential Conflicts of Interest

Nothing to report.

## Supporting information


**Supplementary table 1** information on variant sets used to assess the effect of a general LDL‐C reduction on Alzheimer's disease (AD) risk
**Supplementary table 2:** information on gene‐specific variant sets used to assess the effects of lipid‐lowering drug targets on AD risk in principal components MR models
**Supplementary table 3:** information on alternate gene‐specific variant sets used to assess the effects of lipid‐lowering drug targets on AD risk in IVW MR models with uncorrelated variants
**Supplementary table 4:** information on gene‐specific variant sets used to assess the effects of lipid‐lowering drug targets on cardiometabolic outcomes
**Supplementary table 5:** information on genome‐wide variants used to assess the effects of lowering circulating PCSK9 on AD and CAD
**Supplementary table 6:** Alternate MR methods for examining gene region variants in relation to AD risk, using two LD‐clumping strategies instead of principal component methodology
