# Supplementary material for: Lipid lowering and Alzheimer disease risk: A mendelian randomization study
Source: Ann Neurol. 2019 Dec 13;87(1):30–9. doi: 10.1002/ana.25642 (PMC6944510; doi:10.1002/ana.25642)
Supplement: Supplementary file 1 — Supplementary table 1 information on variant sets used to assess the effect of a general LDL‐C reduction on Alzheimer's disease (AD) risk Supplementary table 2: information on gene‐specific variant sets used to assess the effects of lipid‐lowering drug targets on AD risk in principal components MR models Supplementary table 3: information on alternate gene‐specific variant sets used to assess the effects of lipid‐lowering drug targets on AD risk in IVW MR models with uncorrelated variants Supplementary table 4: information on gene‐specific variant sets used to assess the effects of lipid‐lowering drug targets on cardiometabolic outcomes Supplementary table 5: information on genome‐wide variants used to assess the effects of lowering circulating PCSK9 on AD and CAD Supplementary table 6: Alternate MR methods for examining gene region variants in relation to AD risk, using two LD‐clumping strategies instead of principal component methodology [file ANA-87-30-s001.docx]

**Lipid lowering and Alzheimer’s disease risk: a Mendelian randomization study**

Dylan M. Williams, PhD; Chris Finan, PhD; Amand F. Schmidt, PhD; Stephen Burgess, PhD; Aroon D. Hingorani PhD, FRCP^.^

**Supplementary tables**

Contents

[Supplementary table 1: information on variant sets used to assess the effect of a general LDL-C reduction on Alzheimer's disease (AD) risk 2](#_Toc23319701)

[Supplementary table 2: information on gene-specific variant sets used to assess the effects of lipid-lowering drug targets on AD risk in principal components MR models 7](#_Toc23319702)

[Supplementary table 3: information on alternate gene-specific variant sets used to assess the effects of lipid-lowering drug targets on AD risk in IVW MR models with uncorrelated variants 14](#_Toc23319703)

[Supplementary table 4: information on gene-specific variant sets used to assess the effects of lipid-lowering drug targets on cardiometabolic outcomes 17](#_Toc23319704)

[Supplementary table 5: information on genome-wide variants used to assess the effects of lowering circulating PCSK9 on AD and CAD 22](#_Toc23319705)

[Supplementary table 6: Alternate MR methods for examining gene region variants in relation to AD risk, using two LD-clumping strategies instead of principal component methodology 23](#_Toc23319706)

# Supplementary table 1: information on variant sets used to assess the effect of a general LDL-C reduction on Alzheimer's disease (AD) risk

| **Study** | **SNP** | **Chr** | **Position** | **Effect allele** | **Alt allele** | **EAF exposure** | **Beta exposure** | **SE exposure** | **P exposure** | **Beta outcome** | **SE outcome** | **P outcome** | **Sample size exposure** | **Study exposure** | **Functional**  **consequence** |
| --- | --- | --- | --- | --- | --- | --- | --- | --- | --- | --- | --- | --- | --- | --- | --- |
| IGAP_2019 | rs10102164 | 8 | 55421614 | A | G | 0.201 | 0.031 | 0.003 | 2.58E-21 | 0.015 | 0.018 | 4.05E-01 | 295826 | glgc_exome | intergenic_variant |
| IGAP_2019 | rs1016988 | 5 | 131744574 | C | T | 0.220 | -0.020 | 0.003 | 7.81E-10 | -0.013 | 0.018 | 4.68E-01 | 295826 | glgc_exome | upstream_gene_variant |
| IGAP_2019 | rs10401969 | 19 | 19407718 | C | T | 0.084 | -0.090 | 0.005 | 1.00E-200 | 0.024 | 0.028 | 3.85E-01 | 295826 | glgc_exome | intron_variant |
| IGAP_2019 | rs10490626 | 2 | 118835841 | A | G | 0.069 | -0.053 | 0.005 | 1.29E-22 | -0.057 | 0.027 | 3.29E-02 | 274383 | glgc_exome | intergenic_variant |
| IGAP_2019 | rs10885997 | 10 | 118397971 | G | A | 0.410 | 0.015 | 0.003 | 8.91E-08 | -0.022 | 0.015 | 1.23E-01 | 258146 | glgc_exome | missense_variant |
| IGAP_2019 | rs11065987 | 12 | 112072424 | G | A | 0.364 | -0.026 | 0.003 | 8.01E-19 | -0.006 | 0.015 | 6.98E-01 | 276356 | glgc_exome | intergenic_variant |
| IGAP_2019 | rs11136343 | 8 | 145058986 | G | A | 0.383 | 0.029 | 0.003 | 6.58E-26 | 0.020 | 0.015 | 1.78E-01 | 289480 | glgc_exome | missense_variant |
| IGAP_2019 | rs11220462 | 11 | 126243952 | A | G | 0.145 | 0.043 | 0.005 | 3.64E-21 | 0.017 | 0.021 | 4.16E-01 | 201852 | glgc_exome | intron_variant |
| IGAP_2019 | rs1169288 | 12 | 121416650 | C | A | 0.331 | 0.037 | 0.003 | 1.84E-35 | -0.007 | 0.016 | 6.44E-01 | 271307 | glgc_exome | missense_variant |
| IGAP_2019 | rs12208357 | 6 | 160543148 | T | C | 0.062 | 0.058 | 0.006 | 6.35E-26 | -0.055 | 0.028 | 4.93E-02 | 287807 | glgc_exome | missense_variant |
| IGAP_2019 | rs1250229 | 2 | 216304384 | C | T | 0.741 | 0.015 | 0.003 | 5.46E-07 | -0.006 | 0.017 | 7.32E-01 | 295826 | glgc_exome | upstream_gene_variant |
| IGAP_2019 | rs12670798 | 7 | 21607352 | C | T | 0.246 | 0.033 | 0.003 | 3.47E-25 | -0.001 | 0.017 | 9.58E-01 | 276356 | glgc_exome | intron_variant |
| IGAP_2019 | rs12748152 | 1 | 27138393 | T | C | 0.072 | 0.031 | 0.005 | 2.29E-09 | 0.021 | 0.027 | 4.36E-01 | 292592 | glgc_exome | upstream_gene_variant |
| IGAP_2019 | rs1564348 | 6 | 160578860 | C | T | 0.153 | 0.047 | 0.004 | 2.11E-38 | 0.004 | 0.020 | 8.42E-01 | 295826 | glgc_exome | intron_variant |
| IGAP_2019 | rs17404153 | 3 | 132163200 | T | G | 0.127 | -0.018 | 0.004 | 7.26E-06 | 0.005 | 0.021 | 8.02E-01 | 283592 | glgc_exome | intron_variant |
| IGAP_2019 | rs174546 | 11 | 61569830 | T | C | 0.313 | -0.053 | 0.003 | 1.00E-200 | -0.012 | 0.015 | 4.12E-01 | 293792 | glgc_exome | 3_prime_UTR_variant |
| IGAP_2019 | rs1800562 | 6 | 26093141 | A | G | 0.049 | -0.044 | 0.006 | 2.61E-12 | -0.006 | 0.030 | 8.36E-01 | 291354 | glgc_exome | missense_variant |
| IGAP_2019 | rs1801689 | 17 | 64210580 | C | A | 0.027 | 0.105 | 0.008 | 1.97E-37 | -0.057 | 0.047 | 2.19E-01 | 295826 | glgc_exome | missense_variant |
| IGAP_2019 | rs1891110 | 10 | 124610027 | A | G | 0.548 | 0.021 | 0.003 | 7.69E-15 | -0.004 | 0.014 | 7.72E-01 | 295826 | glgc_exome | missense_variant |
| IGAP_2019 | rs2000999 | 16 | 72108093 | A | G | 0.209 | 0.063 | 0.004 | 1.00E-200 | -0.024 | 0.018 | 1.80E-01 | 262517 | glgc_exome | intron_variant |
| IGAP_2019 | rs2030746 | 2 | 121309488 | T | C | 0.413 | 0.014 | 0.003 | 1.88E-07 | -0.011 | 0.015 | 4.56E-01 | 295826 | glgc_exome | downstream_gene_variant |
| IGAP_2019 | rs2076674 | 22 | 41170063 | C | T | 0.354 | 0.018 | 0.003 | 8.08E-11 | -0.005 | 0.015 | 7.45E-01 | 285068 | glgc_exome | intron_variant |
| IGAP_2019 | rs2081687 | 8 | 59388565 | C | T | 0.663 | -0.028 | 0.003 | 3.76E-23 | 0.001 | 0.015 | 9.33E-01 | 295826 | glgc_exome | intergenic_variant |
| IGAP_2019 | rs2125345 | 17 | 73782191 | C | T | 0.337 | -0.024 | 0.003 | 4.74E-16 | -0.003 | 0.016 | 8.63E-01 | 276799 | glgc_exome | intron_variant |
| IGAP_2019 | rs2131925 | 1 | 63025942 | T | G | 0.648 | 0.044 | 0.003 | 1.00E-200 | -0.023 | 0.015 | 1.23E-01 | 295302 | glgc_exome | intron_variant |
| IGAP_2019 | rs2239619 | 6 | 52453220 | A | C | 0.619 | 0.018 | 0.003 | 7.42E-11 | 0.012 | 0.015 | 4.20E-01 | 284338 | glgc_exome | downstream_gene_variant |
| IGAP_2019 | rs2255141 | 10 | 113933886 | G | A | 0.728 | -0.028 | 0.003 | 7.68E-21 | -0.009 | 0.016 | 5.50E-01 | 295826 | glgc_exome | intron_variant |
| IGAP_2019 | rs2328223 | 20 | 17845921 | C | A | 0.249 | 0.030 | 0.005 | 5.63E-09 | 0.037 | 0.018 | 4.16E-02 | 170762 | glgc_gwas | intergenic_variant |
| IGAP_2019 | rs267733 | 1 | 150958836 | G | A | 0.137 | -0.025 | 0.004 | 5.59E-11 | -0.025 | 0.020 | 2.11E-01 | 294565 | glgc_exome | missense_variant |
| IGAP_2019 | rs28929474 | 14 | 94844947 | T | C | 0.015 | 0.081 | 0.011 | 4.30E-14 | -0.068 | 0.056 | 2.27E-01 | 290263 | glgc_exome | missense_variant |
| IGAP_2019 | rs314253 | 17 | 7091650 | C | T | 0.351 | -0.020 | 0.003 | 1.10E-12 | 0.007 | 0.015 | 6.53E-01 | 295826 | glgc_exome | downstream_gene_variant |
| IGAP_2019 | rs3177928 | 6 | 32412435 | A | G | 0.138 | 0.035 | 0.004 | 6.10E-17 | 0.073 | 0.021 | 5.23E-04 | 253199 | glgc_exome | 3_prime_UTR_variant |
| IGAP_2019 | rs351855 | 5 | 176520243 | A | G | 0.289 | -0.018 | 0.003 | 3.94E-08 | -0.021 | 0.016 | 1.90E-01 | 233058 | glgc_exome | missense_variant |
| IGAP_2019 | rs364585 | 20 | 12962718 | G | A | 0.639 | 0.019 | 0.003 | 3.99E-11 | -0.016 | 0.015 | 2.67E-01 | 276356 | glgc_exome | intergenic_variant |
| IGAP_2019 | rs3764261 | 16 | 56993324 | A | C | 0.313 | -0.032 | 0.003 | 1.48E-29 | -0.003 | 0.015 | 8.35E-01 | 289809 | glgc_exome | upstream_gene_variant |
| IGAP_2019 | rs3780181 | 9 | 2640759 | G | A | 0.075 | -0.037 | 0.005 | 1.80E-13 | -0.035 | 0.030 | 2.47E-01 | 295826 | glgc_exome | intron_variant |
| IGAP_2019 | rs3812594 | 9 | 139368953 | A | G | 0.238 | -0.018 | 0.003 | 1.54E-08 | -0.019 | 0.016 | 2.26E-01 | 293723 | glgc_exome | missense_variant |
| IGAP_2019 | rs4253772 | 22 | 46627603 | T | C | 0.096 | 0.018 | 0.005 | 0.0001014 | 0.021 | 0.023 | 3.66E-01 | 276356 | glgc_exome | intron_variant |
| IGAP_2019 | rs4530754 | 5 | 122855416 | A | G | 0.556 | 0.017 | 0.003 | 9.50E-10 | 0.024 | 0.014 | 9.08E-02 | 276356 | glgc_exome | intron_variant |
| IGAP_2019 | rs4722551 | 7 | 25991826 | C | T | 0.158 | 0.040 | 0.004 | 2.13E-26 | -0.016 | 0.020 | 4.13E-01 | 275110 | glgc_exome | upstream_gene_variant |
| IGAP_2019 | rs4942486 | 13 | 32953388 | C | T | 0.516 | -0.022 | 0.003 | 4.48E-16 | 0.007 | 0.014 | 6.04E-01 | 291053 | glgc_exome | intron_variant |
| IGAP_2019 | rs5763662 | 22 | 30378703 | T | C | 0.042 | 0.025 | 0.008 | 0.001783 | 0.013 | 0.048 | 7.85E-01 | 265024 | glgc_exome | intron_variant |
| IGAP_2019 | rs61754230 | 12 | 72179446 | T | C | 0.016 | 0.057 | 0.011 | 1.14E-07 | -0.055 | 0.083 | 5.11E-01 | 292762 | glgc_exome | missense_variant |
| IGAP_2019 | rs629301 | 1 | 109818306 | T | G | 0.774 | 0.158 | 0.003 | 1.00E-200 | 0.008 | 0.017 | 6.28E-01 | 295826 | glgc_exome | 3_prime_UTR_variant |
| IGAP_2019 | rs6511720 | 19 | 11202306 | T | G | 0.109 | -0.211 | 0.004 | 1.00E-200 | -0.010 | 0.022 | 6.60E-01 | 295826 | glgc_exome | intron_variant |
| IGAP_2019 | rs6756629 | 2 | 44065090 | A | G | 0.063 | -0.111 | 0.005 | 1.00E-200 | 0.033 | 0.029 | 2.56E-01 | 295826 | glgc_exome | missense_variant |
| IGAP_2019 | rs6831256 | 4 | 3473139 | G | A | 0.442 | 0.013 | 0.003 | 2.28E-06 | -0.002 | 0.015 | 8.67E-01 | 291053 | glgc_exome | intron_variant |
| IGAP_2019 | rs6882076 | 5 | 156390297 | C | T | 0.623 | 0.039 | 0.003 | 1.00E-200 | 0.008 | 0.015 | 5.77E-01 | 295826 | glgc_exome | upstream_gene_variant |
| IGAP_2019 | rs704 | 17 | 26694861 | A | G | 0.488 | 0.021 | 0.003 | 5.58E-16 | -0.001 | 0.015 | 9.71E-01 | 295826 | glgc_exome | missense_variant |
| IGAP_2019 | rs7640978 | 3 | 32533010 | T | C | 0.094 | -0.033 | 0.005 | 6.15E-13 | 0.003 | 0.025 | 9.09E-01 | 295826 | glgc_exome | intron_variant |
| IGAP_2019 | rs8017377 | 14 | 24883887 | A | G | 0.425 | 0.023 | 0.003 | 6.03E-17 | 0.003 | 0.015 | 8.61E-01 | 295826 | glgc_exome | missense_variant |
| IGAP_2019 | rs9370867 | 6 | 16145325 | G | A | 0.530 | -0.033 | 0.003 | 3.02E-34 | 0.018 | 0.014 | 2.13E-01 | 290263 | glgc_exome | missense_variant |
| IGAP_2019 | rs9646133 | 14 | 71096344 | T | G | 0.326 | -0.019 | 0.003 | 2.35E-11 | 0.001 | 0.016 | 9.35E-01 | 295826 | glgc_exome | intron_variant |
| IGAP_2019 | rs9987289 | 8 | 9183358 | G | A | 0.899 | 0.057 | 0.005 | 4.28E-36 | -0.027 | 0.026 | 2.92E-01 | 278685 | glgc_exome | intron_variant |
| PGC | rs10102164 | 8 | 55421614 | A | G | 0.201 | 0.031 | 0.003 | 2.58E-21 | 0.006 | 0.045 | 8.96E-01 | 295826 | glgc_exome | intergenic_variant |
| PGC | rs1016988 | 5 | 131744574 | C | T | 0.220 | -0.020 | 0.003 | 7.81E-10 | -0.065 | 0.048 | 1.69E-01 | 295826 | glgc_exome | upstream_gene_variant |
| PGC | rs10401969 | 19 | 19407718 | C | T | 0.084 | -0.090 | 0.005 | 1.00E-200 | 0.105 | 0.063 | 9.49E-02 | 295826 | glgc_exome | intron_variant |
| PGC | rs10490626 | 2 | 118835841 | A | G | 0.069 | -0.053 | 0.005 | 1.29E-22 | 0.073 | 0.075 | 3.31E-01 | 274383 | glgc_exome | intergenic_variant |
| PGC | rs10885997 | 10 | 118397971 | G | A | 0.410 | 0.015 | 0.003 | 8.91E-08 | -0.047 | 0.038 | 2.16E-01 | 258146 | glgc_exome | missense_variant |
| PGC | rs11065987 | 12 | 112072424 | G | A | 0.364 | -0.026 | 0.003 | 8.01E-19 | -0.032 | 0.039 | 4.14E-01 | 276356 | glgc_exome | intergenic_variant |
| PGC | rs11136343 | 8 | 145058986 | G | A | 0.383 | 0.029 | 0.003 | 6.58E-26 | 0.062 | 0.040 | 1.20E-01 | 289480 | glgc_exome | missense_variant |
| PGC | rs11220462 | 11 | 126243952 | A | G | 0.145 | 0.043 | 0.005 | 3.64E-21 | 0.045 | 0.055 | 4.13E-01 | 201852 | glgc_exome | intron_variant |
| PGC | rs1169288 | 12 | 121416650 | C | A | 0.331 | 0.037 | 0.003 | 1.84E-35 | -0.006 | 0.041 | 8.82E-01 | 271307 | glgc_exome | missense_variant |
| PGC | rs12208357 | 6 | 160543148 | T | C | 0.062 | 0.058 | 0.006 | 6.35E-26 | 0.053 | 0.071 | 4.56E-01 | 287807 | glgc_exome | missense_variant |
| PGC | rs1250229 | 2 | 216304384 | C | T | 0.741 | 0.015 | 0.003 | 5.46E-07 | 0.111 | 0.044 | 1.14E-02 | 295826 | glgc_exome | upstream_gene_variant |
| PGC | rs12670798 | 7 | 21607352 | C | T | 0.246 | 0.033 | 0.003 | 3.47E-25 | -0.029 | 0.043 | 4.94E-01 | 276356 | glgc_exome | intron_variant |
| PGC | rs12748152 | 1 | 27138393 | T | C | 0.072 | 0.031 | 0.005 | 2.29E-09 | -0.037 | 0.065 | 5.67E-01 | 292592 | glgc_exome | upstream_gene_variant |
| PGC | rs1564348 | 6 | 160578860 | C | T | 0.153 | 0.047 | 0.004 | 2.11E-38 | -0.059 | 0.053 | 2.64E-01 | 295826 | glgc_exome | intron_variant |
| PGC | rs17404153 | 3 | 132163200 | T | G | 0.127 | -0.018 | 0.004 | 7.26E-06 | 0.043 | 0.062 | 4.91E-01 | 283592 | glgc_exome | intron_variant |
| PGC | rs174546 | 11 | 61569830 | T | C | 0.313 | -0.053 | 0.003 | 1.00E-200 | -0.068 | 0.040 | 8.57E-02 | 293792 | glgc_exome | 3_prime_UTR_variant |
| PGC | rs1800562 | 6 | 26093141 | A | G | 0.049 | -0.044 | 0.006 | 2.61E-12 | 0.000 | 0.075 | 1.00E+00 | 291354 | glgc_exome | missense_variant |
| PGC | rs1801689 | 17 | 64210580 | C | A | 0.027 | 0.105 | 0.008 | 1.97E-37 | -0.084 | 0.168 | 6.17E-01 | 295826 | glgc_exome | missense_variant |
| PGC | rs1891110 | 10 | 124610027 | A | G | 0.548 | 0.021 | 0.003 | 7.69E-15 | 0.061 | 0.038 | 1.13E-01 | 295826 | glgc_exome | missense_variant |
| PGC | rs2000999 | 16 | 72108093 | A | G | 0.209 | 0.063 | 0.004 | 1.00E-200 | 0.011 | 0.047 | 8.21E-01 | 262517 | glgc_exome | intron_variant |
| PGC | rs2030746 | 2 | 121309488 | T | C | 0.413 | 0.014 | 0.003 | 1.88E-07 | -0.008 | 0.038 | 8.35E-01 | 295826 | glgc_exome | downstream_gene_variant |
| PGC | rs2076674 | 22 | 41170063 | C | T | 0.354 | 0.018 | 0.003 | 8.08E-11 | -0.022 | 0.040 | 5.81E-01 | 285068 | glgc_exome | intron_variant |
| PGC | rs2081687 | 8 | 59388565 | C | T | 0.663 | -0.028 | 0.003 | 3.76E-23 | 0.034 | 0.040 | 4.07E-01 | 295826 | glgc_exome | intergenic_variant |
| PGC | rs2125345 | 17 | 73782191 | C | T | 0.337 | -0.024 | 0.003 | 4.74E-16 | -0.012 | 0.044 | 7.76E-01 | 276799 | glgc_exome | intron_variant |
| PGC | rs2131925 | 1 | 63025942 | T | G | 0.648 | 0.044 | 0.003 | 1.00E-200 | -0.009 | 0.040 | 8.16E-01 | 295302 | glgc_exome | intron_variant |
| PGC | rs2239619 | 6 | 52453220 | A | C | 0.619 | 0.018 | 0.003 | 7.42E-11 | 0.052 | 0.039 | 1.88E-01 | 284338 | glgc_exome | downstream_gene_variant |
| PGC | rs2255141 | 10 | 113933886 | G | A | 0.728 | -0.028 | 0.003 | 7.68E-21 | 0.009 | 0.043 | 8.30E-01 | 295826 | glgc_exome | intron_variant |
| PGC | rs2328223 | 20 | 17845921 | C | A | 0.249 | 0.030 | 0.005 | 5.63E-09 | 0.026 | 0.052 | 6.10E-01 | 170762 | glgc_gwas | intergenic_variant |
| PGC | rs267733 | 1 | 150958836 | G | A | 0.137 | -0.025 | 0.004 | 5.59E-11 | -0.061 | 0.053 | 2.52E-01 | 294565 | glgc_exome | missense_variant |
| PGC | rs28929474 | 14 | 94844947 | T | C | 0.015 | 0.081 | 0.011 | 4.30E-14 | 0.272 | 0.131 | 3.74E-02 | 290263 | glgc_exome | missense_variant |
| PGC | rs314253 | 17 | 7091650 | C | T | 0.351 | -0.020 | 0.003 | 1.10E-12 | -0.030 | 0.039 | 4.39E-01 | 295826 | glgc_exome | downstream_gene_variant |
| PGC | rs3177928 | 6 | 32412435 | A | G | 0.138 | 0.035 | 0.004 | 6.10E-17 | 0.083 | 0.054 | 1.25E-01 | 253199 | glgc_exome | 3_prime_UTR_variant |
| PGC | rs351855 | 5 | 176520243 | A | G | 0.289 | -0.018 | 0.003 | 3.94E-08 | -0.018 | 0.042 | 6.73E-01 | 233058 | glgc_exome | missense_variant |
| PGC | rs364585 | 20 | 12962718 | G | A | 0.639 | 0.019 | 0.003 | 3.99E-11 | -0.054 | 0.039 | 1.62E-01 | 276356 | glgc_exome | intergenic_variant |
| PGC | rs3764261 | 16 | 56993324 | A | C | 0.313 | -0.032 | 0.003 | 1.48E-29 | 0.017 | 0.040 | 6.74E-01 | 289809 | glgc_exome | upstream_gene_variant |
| PGC | rs3780181 | 9 | 2640759 | G | A | 0.075 | -0.037 | 0.005 | 1.80E-13 | -0.025 | 0.080 | 7.56E-01 | 295826 | glgc_exome | intron_variant |
| PGC | rs3812594 | 9 | 139368953 | A | G | 0.238 | -0.018 | 0.003 | 1.54E-08 | -0.038 | 0.043 | 3.80E-01 | 293723 | glgc_exome | missense_variant |
| PGC | rs4253772 | 22 | 46627603 | T | C | 0.096 | 0.018 | 0.005 | 0.0001014 | -0.005 | 0.066 | 9.43E-01 | 276356 | glgc_exome | intron_variant |
| PGC | rs4530754 | 5 | 122855416 | A | G | 0.556 | 0.017 | 0.003 | 9.50E-10 | 0.086 | 0.038 | 2.44E-02 | 276356 | glgc_exome | intron_variant |
| PGC | rs4722551 | 7 | 25991826 | C | T | 0.158 | 0.040 | 0.004 | 2.13E-26 | -0.097 | 0.052 | 6.08E-02 | 275110 | glgc_exome | upstream_gene_variant |
| PGC | rs4942486 | 13 | 32953388 | C | T | 0.516 | -0.022 | 0.003 | 4.48E-16 | -0.034 | 0.038 | 3.75E-01 | 291053 | glgc_exome | intron_variant |
| PGC | rs5763662 | 22 | 30378703 | T | C | 0.042 | 0.025 | 0.008 | 0.001783 | 0.067 | 0.143 | 6.38E-01 | 265024 | glgc_exome | intron_variant |
| PGC | rs61754230 | 12 | 72179446 | T | C | 0.016 | 0.057 | 0.011 | 1.14E-07 | -0.136 | 0.270 | 6.15E-01 | 292762 | glgc_exome | missense_variant |
| PGC | rs629301 | 1 | 109818306 | T | G | 0.774 | 0.158 | 0.003 | 1.00E-200 | -0.013 | 0.045 | 7.78E-01 | 295826 | glgc_exome | 3_prime_UTR_variant |
| PGC | rs6511720 | 19 | 11202306 | T | G | 0.109 | -0.211 | 0.004 | 1.00E-200 | 0.054 | 0.063 | 3.89E-01 | 295826 | glgc_exome | intron_variant |
| PGC | rs6756629 | 2 | 44065090 | A | G | 0.063 | -0.111 | 0.005 | 1.00E-200 | 0.065 | 0.076 | 3.92E-01 | 295826 | glgc_exome | missense_variant |
| PGC | rs6831256 | 4 | 3473139 | G | A | 0.442 | 0.013 | 0.003 | 2.28E-06 | 0.039 | 0.038 | 3.02E-01 | 291053 | glgc_exome | intron_variant |
| PGC | rs6882076 | 5 | 156390297 | C | T | 0.623 | 0.039 | 0.003 | 1.00E-200 | 0.094 | 0.040 | 1.81E-02 | 295826 | glgc_exome | upstream_gene_variant |
| PGC | rs704 | 17 | 26694861 | A | G | 0.488 | 0.021 | 0.003 | 5.58E-16 | -0.002 | 0.039 | 9.63E-01 | 295826 | glgc_exome | missense_variant |
| PGC | rs7640978 | 3 | 32533010 | T | C | 0.094 | -0.033 | 0.005 | 6.15E-13 | 0.154 | 0.073 | 3.55E-02 | 295826 | glgc_exome | intron_variant |
| PGC | rs8017377 | 14 | 24883887 | A | G | 0.425 | 0.023 | 0.003 | 6.03E-17 | -0.005 | 0.038 | 8.90E-01 | 295826 | glgc_exome | missense_variant |
| PGC | rs9370867 | 6 | 16145325 | G | A | 0.530 | -0.033 | 0.003 | 3.02E-34 | 0.040 | 0.038 | 2.94E-01 | 290263 | glgc_exome | missense_variant |
| PGC | rs964184 | 11 | 116648917 | C | G | 0.847 | -0.037 | 0.004 | 1.13E-23 | -0.045 | 0.057 | 4.29E-01 | 295826 | glgc_exome | 3_prime_UTR_variant |
| PGC | rs9646133 | 14 | 71096344 | T | G | 0.326 | -0.019 | 0.003 | 2.35E-11 | 0.073 | 0.043 | 8.80E-02 | 295826 | glgc_exome | intron_variant |
| PGC | rs9987289 | 8 | 9183358 | G | A | 0.899 | 0.057 | 0.005 | 4.28E-36 | -0.017 | 0.062 | 7.82E-01 | 278685 | glgc_exome | intron_variant |

*Abbreviations: Chr – chromosome; EAF - effect allele frequency; glgc_exome - Global Lipid Genetics Consortium exome-wide study; glgc_gwas - Global Lipid Genetics Consortium genome-wide association study; IGAP - International Genomics of Alzheimer's disease project; PGC - Psychiatric Genomics Consortium; SNP - single nucleotide polymorphism*

Foot notes:

1) SNP positions are from human reference genome build GRChr37

2) P values for association with LDL-C were recorded with a lower limit of 1.00E-200

3) Some P values for SNP-LDL associations are not below genome-wide significance threshold (5e-08): these are either discovery sample P values that were replicated in additional data in the GLGC exome-wide study, or exome-wide results that had been reported as having P values under 5e-08 in the previous GLGC GWAS (in which case, exome-wide estimates were prioritised due to these being derived from larger sample sizes)

4) Functional annotations for SNPs were derived from Ensembl Variant Effect Predictor

# Supplementary table 2: information on gene-specific variant sets used to assess the effects of lipid-lowering drug targets on AD risk in principal components MR models

| **Study** | **Gene** | **SNP** | **Chr** | **Position** | **Effect allele** | **Alt allele** | **EAF exposure** | **Beta exposure** | **SE exposure** | **P exposure** | **Beta outcome** | **SE outcome** | **P outcome** | **Sample size exposure** | **Study exposure** | **Functional**  **consequence** |
| --- | --- | --- | --- | --- | --- | --- | --- | --- | --- | --- | --- | --- | --- | --- | --- | --- |
| IGAP_2019 | APOB | rs10199768 | 2 | 21244000 | T | G | 0.385 | 0.097 | 0.004 | 1.00E-200 | -0.005 | 0.015 | 7.54E-01 | 170875 | glgc_gwas | intron_variant |
| IGAP_2019 | APOB | rs1042031 | 2 | 21225753 | T | C | 0.172 | -0.045 | 0.004 | 3.04E-37 | 0.030 | 0.018 | 1.04E-01 | 295826 | glgc_exome | stop_gained |
| IGAP_2019 | APOB | rs11676704 | 2 | 21244358 | T | G | 0.844 | 0.001 | 0.005 | 6.16E-01 | 0.040 | 0.021 | 5.07E-02 | 173023 | glgc_gwas | intron_variant |
| IGAP_2019 | APOB | rs12691202 | 2 | 21249716 | C | T | 0.949 | 0.097 | 0.011 | 8.22E-19 | -0.030 | 0.043 | 4.75E-01 | 109096 | glgc_gwas | missense_variant |
| IGAP_2019 | APOB | rs12713771 | 2 | 21235565 | T | C | 0.018 | 0.022 | 0.037 | 5.53E-01 | 0.023 | 0.104 | 8.24E-01 | 21554 | magnetic | intron_variant |
| IGAP_2019 | APOB | rs12713870 | 2 | 21239595 | G | A | 0.022 | 0.036 | 0.037 | 3.34E-01 | -0.089 | 0.051 | 7.85E-02 | 21553 | magnetic | intron_variant |
| IGAP_2019 | APOB | rs12720796 | 2 | 21261998 | C | A | 0.022 | 0.091 | 0.014 | 1.68E-10 | -0.003 | 0.061 | 9.57E-01 | 153714 | glgc_gwas | intron_variant |
| IGAP_2019 | APOB | rs12720816 | 2 | 21239667 | C | T | 0.329 | 0.090 | 0.011 | 1.02E-16 | -0.013 | 0.015 | 3.81E-01 | 21556 | magnetic | intron_variant |
| IGAP_2019 | APOB | rs12720842 | 2 | 21257927 | C | T | 0.021 | 0.099 | 0.012 | 1.88E-15 | -0.020 | 0.043 | 6.45E-01 | 161300 | glgc_gwas | intron_variant |
| IGAP_2019 | APOB | rs1367117 | 2 | 21263900 | A | G | 0.285 | 0.105 | 0.003 | 1.00E-200 | 0.004 | 0.016 | 7.94E-01 | 295826 | glgc_exome | missense_variant |
| IGAP_2019 | APOB | rs149790267 | 2 | 21224120 | G | A | 0.018 | 0.020 | 0.039 | 6.12E-01 | 0.018 | 0.066 | 7.87E-01 | 21555 | magnetic | upstream_gene_variant |
| IGAP_2019 | APOB | rs150956240 | 2 | 21238984 | G | A | 0.010 | 0.000 | 0.056 | 9.94E-01 | 0.004 | 0.071 | 9.57E-01 | 21554 | magnetic | intron_variant |
| IGAP_2019 | APOB | rs1799812 | 2 | 21229609 | T | C | 0.008 | 0.026 | 0.059 | 6.62E-01 | -0.081 | 0.105 | 4.41E-01 | 19269 | magnetic | synonymous_variant |
| IGAP_2019 | APOB | rs1801695 | 2 | 21224853 | C | T | 0.974 | 0.002 | 0.014 | 7.41E-01 | -0.037 | 0.047 | 4.32E-01 | 105182 | glgc_gwas | missense_variant |
| IGAP_2019 | APOB | rs1801699 | 2 | 21233999 | C | T | 0.018 | 0.075 | 0.010 | 8.56E-14 | -0.026 | 0.054 | 6.30E-01 | 273292 | glgc_exome | missense_variant |
| IGAP_2019 | APOB | rs1801700 | 2 | 21245813 | A | G | 0.042 | 0.018 | 0.008 | 9.56E-02 | 0.048 | 0.035 | 1.71E-01 | 168112 | glgc_gwas | missense_variant |
| IGAP_2019 | APOB | rs1801701 | 2 | 21228827 | T | C | 0.081 | 0.046 | 0.005 | 4.87E-21 | -0.017 | 0.024 | 4.91E-01 | 293853 | glgc_exome | missense_variant |
| IGAP_2019 | APOB | rs184507838 | 2 | 21255764 | T | C | 0.031 | -0.099 | 0.029 | 6.12E-04 | -0.056 | 0.079 | 4.76E-01 | 21553 | magnetic | intron_variant |
| IGAP_2019 | APOB | rs2678379 | 2 | 21226560 | G | A | 0.768 | 0.058 | 0.004 | 1.65E-38 | 0.035 | 0.017 | 4.19E-02 | 173015 | glgc_gwas | intron_variant |
| IGAP_2019 | APOB | rs2854725 | 2 | 21237786 | T | G | 0.901 | 0.064 | 0.007 | 4.99E-19 | -0.028 | 0.026 | 2.72E-01 | 172961 | glgc_gwas | intron_variant |
| IGAP_2019 | APOB | rs3791981 | 2 | 21245367 | A | G | 0.880 | 0.094 | 0.007 | 1.00E-200 | -0.043 | 0.024 | 7.62E-02 | 161484 | glgc_gwas | intron_variant |
| IGAP_2019 | APOB | rs512535 | 2 | 21267782 | T | C | 0.484 | 0.031 | 0.004 | 2.32E-15 | 0.023 | 0.015 | 1.14E-01 | 172987 | glgc_gwas | upstream_gene_variant |
| IGAP_2019 | APOB | rs531819 | 2 | 21263639 | G | T | 0.809 | 0.134 | 0.005 | 1.00E-200 | -0.034 | 0.020 | 8.29E-02 | 173033 | glgc_gwas | intron_variant |
| IGAP_2019 | APOB | rs533617 | 2 | 21233972 | C | T | 0.039 | -0.127 | 0.007 | 1.00E-200 | -0.019 | 0.039 | 6.26E-01 | 291354 | glgc_exome | missense_variant |
| IGAP_2019 | APOB | rs6413458 | 2 | 21231592 | G | A | 0.976 | 0.094 | 0.014 | 2.19E-10 | -0.060 | 0.055 | 2.77E-01 | 159056 | glgc_gwas | synonymous_variant |
| IGAP_2019 | APOB | rs679899 | 2 | 21250914 | A | G | 0.478 | -0.045 | 0.003 | 1.00E-200 | -0.016 | 0.014 | 2.67E-01 | 295826 | glgc_exome | missense_variant |
| IGAP_2019 | APOB | rs72653053 | 2 | 21266223 | T | C | 0.983 | 0.100 | 0.028 | 4.47E-03 | -0.046 | 0.068 | 5.01E-01 | 75509 | glgc_gwas | intron_variant |
| IGAP_2019 | HMGCR | rs10045497 | 5 | 74636484 | A | C | 0.408 | 0.077 | 0.005 | 1.00E-200 | 0.001 | 0.015 | 9.38E-01 | 89888 | glgc_gwas | intron_variant |
| IGAP_2019 | HMGCR | rs10474434 | 5 | 74644681 | T | G | 0.252 | 0.063 | 0.006 | 4.11E-22 | 0.042 | 0.017 | 1.42E-02 | 83136 | glgc_gwas | intron_variant |
| IGAP_2019 | HMGCR | rs10474435 | 5 | 74657280 | C | T | 0.009 | 0.054 | 0.015 | 2.36E-03 | 0.165 | 0.065 | 1.14E-02 | 150792 | glgc_gwas | 3_prime_UTR_variant |
| IGAP_2019 | HMGCR | rs10515198 | 5 | 74641560 | A | G | 0.103 | 0.060 | 0.006 | 5.99E-22 | -0.049 | 0.024 | 4.10E-02 | 173012 | glgc_gwas | intron_variant |
| IGAP_2019 | HMGCR | rs142550951 | 5 | 74649285 | T | C | 0.051 | 0.040 | 0.023 | 8.86E-02 | -0.020 | 0.033 | 5.38E-01 | 21554 | magnetic | intron_variant |
| IGAP_2019 | HMGCR | rs142563098 | 5 | 74633014 | C | T | 0.006 | 0.022 | 0.107 | 8.43E-01 | -0.102 | 0.099 | 3.04E-01 | 14004 | magnetic | 5_prime_UTR_variant |
| IGAP_2019 | HMGCR | rs17238540 | 5 | 74655498 | T | G | 0.979 | 0.024 | 0.016 | 1.58E-01 | -0.016 | 0.044 | 7.20E-01 | 84983 | glgc_gwas | non_coding_transcript_exon_variant |
| IGAP_2019 | HMGCR | rs17238596 | 5 | 74652059 | T | C | 0.047 | -0.081 | 0.028 | 5.08E-03 | 0.033 | 0.049 | 5.03E-01 | 19269 | magnetic | intron_variant |
| IGAP_2019 | HMGCR | rs17244792 | 5 | 74640625 | A | G | 0.023 | -0.041 | 0.040 | 3.09E-01 | 0.045 | 0.079 | 5.73E-01 | 19270 | magnetic | intron_variant |
| IGAP_2019 | HMGCR | rs17648121 | 5 | 74650106 | T | C | 0.045 | 0.054 | 0.026 | 3.98E-02 | 0.044 | 0.046 | 3.42E-01 | 19266 | magnetic | intron_variant |
| IGAP_2019 | HMGCR | rs2303151 | 5 | 74655451 | T | C | 0.054 | 0.015 | 0.008 | 2.20E-02 | -0.052 | 0.032 | 1.05E-01 | 170080 | glgc_gwas | non_coding_transcript_exon_variant |
| IGAP_2019 | HMGCR | rs2303152 | 5 | 74641707 | A | G | 0.120 | 0.042 | 0.006 | 1.04E-09 | 0.017 | 0.023 | 4.55E-01 | 160116 | glgc_gwas | intron_variant |
| IGAP_2019 | HMGCR | rs5908 | 5 | 74652199 | G | A | 0.018 | -0.036 | 0.010 | 2.32E-04 | 0.007 | 0.075 | 9.31E-01 | 290263 | glgc_exome | missense_variant |
| IGAP_2019 | HMGCR | rs76475757 | 5 | 74637213 | C | T | 0.061 | 0.056 | 0.010 | 4.88E-07 | -0.033 | 0.031 | 2.89E-01 | 83142 | glgc_gwas | intron_variant |
| IGAP_2019 | NPC1L1 | rs10264715 | 7 | 44555406 | A | G | 0.199 | 0.022 | 0.004 | 1.65E-06 | -0.020 | 0.017 | 2.51E-01 | 173026 | glgc_gwas | synonymous_variant |
| IGAP_2019 | NPC1L1 | rs113223818 | 7 | 44577569 | A | G | 0.025 | 0.003 | 0.020 | 7.91E-01 | 0.107 | 0.078 | 1.73E-01 | 54559 | glgc_gwas | intron_variant |
| IGAP_2019 | NPC1L1 | rs114376659 | 7 | 44579335 | A | G | 0.008 | -0.045 | 0.014 | 2.00E-03 | 0.008 | 0.088 | 9.28E-01 | 282683 | glgc_exome | missense_variant |
| IGAP_2019 | NPC1L1 | rs117448375 | 7 | 44566246 | T | C | 0.007 | -0.089 | 0.075 | 2.43E-01 | 0.043 | 0.092 | 6.39E-01 | 21556 | magnetic | intron_variant |
| IGAP_2019 | NPC1L1 | rs139264665 | 7 | 44564912 | G | A | 0.019 | 0.059 | 0.046 | 2.06E-01 | -0.024 | 0.058 | 6.78E-01 | 19269 | magnetic | intron_variant |
| IGAP_2019 | NPC1L1 | rs139912571 | 7 | 44565670 | A | C | 0.019 | 0.040 | 0.042 | 3.52E-01 | 0.062 | 0.038 | 9.99E-02 | 21553 | magnetic | intron_variant |
| IGAP_2019 | NPC1L1 | rs142301466 | 7 | 44558216 | T | C | 0.010 | -0.058 | 0.058 | 3.17E-01 | -0.031 | 0.065 | 6.37E-01 | 19267 | magnetic | synonymous_variant |
| IGAP_2019 | NPC1L1 | rs17655652 | 7 | 44580991 | T | C | 0.711 | 0.028 | 0.004 | 2.18E-10 | 0.007 | 0.016 | 6.67E-01 | 162152 | glgc_gwas | upstream_gene_variant |
| IGAP_2019 | NPC1L1 | rs2073548 | 7 | 44581620 | G | A | 0.061 | 0.014 | 0.013 | 4.25E-01 | 0.006 | 0.042 | 8.80E-01 | 68864 | glgc_gwas | upstream_gene_variant |
| IGAP_2019 | NPC1L1 | rs217426 | 7 | 44558859 | A | C | 0.974 | 0.005 | 0.011 | 6.64E-01 | -0.010 | 0.038 | 7.87E-01 | 164788 | glgc_gwas | intron_variant |
| IGAP_2019 | NPC1L1 | rs217432 | 7 | 44553826 | G | A | 0.195 | 0.022 | 0.006 | 5.84E-04 | -0.012 | 0.017 | 4.86E-01 | 89888 | glgc_gwas | intron_variant |
| IGAP_2019 | NPC1L1 | rs35349497 | 7 | 44561635 | C | T | 0.905 | 0.018 | 0.009 | 5.90E-02 | -0.021 | 0.029 | 4.71E-01 | 82303 | glgc_gwas | intron_variant |
| IGAP_2019 | NPC1L1 | rs41279627 | 7 | 44571628 | C | T | 0.954 | 0.026 | 0.013 | 8.03E-02 | -0.001 | 0.042 | 9.81E-01 | 83146 | glgc_gwas | intron_variant |
| IGAP_2019 | NPC1L1 | rs41279633 | 7 | 44580876 | T | G | 0.135 | 0.052 | 0.007 | 2.27E-12 | -0.043 | 0.021 | 3.86E-02 | 80008 | glgc_gwas | 5_prime_UTR_variant |
| IGAP_2019 | NPC1L1 | rs4720470 | 7 | 44561884 | C | T | 0.933 | 0.002 | 0.010 | 9.44E-01 | 0.009 | 0.035 | 7.88E-01 | 117734 | glgc_gwas | intron_variant |
| IGAP_2019 | NPC1L1 | rs56243746 | 7 | 44581150 | G | A | 0.021 | 0.018 | 0.016 | 2.54E-01 | 0.041 | 0.075 | 5.86E-01 | 72546 | glgc_gwas | upstream_gene_variant |
| IGAP_2019 | NPC1L1 | rs62459147 | 7 | 44566917 | T | C | 0.052 | -0.043 | 0.025 | 9.05E-02 | 0.003 | 0.043 | 9.53E-01 | 19267 | magnetic | intron_variant |
| IGAP_2019 | NPC1L1 | rs73107472 | 7 | 44575664 | T | G | 0.013 | 0.009 | 0.022 | 8.73E-01 | 0.030 | 0.062 | 6.36E-01 | 77803 | glgc_gwas | intron_variant |
| IGAP_2019 | NPC1L1 | rs77826622 | 7 | 44572346 | C | T | 0.017 | 0.063 | 0.045 | 1.74E-01 | -0.049 | 0.060 | 4.09E-01 | 21554 | magnetic | intron_variant |
| IGAP_2019 | NPC1L1 | rs7808295 | 7 | 44572699 | T | C | 0.028 | -0.024 | 0.035 | 4.96E-01 | -0.053 | 0.050 | 2.86E-01 | 21554 | magnetic | intron_variant |
| IGAP_2019 | PCSK9 | rs11206514 | 1 | 55516004 | A | C | 0.611 | 0.051 | 0.004 | 9.95E-33 | -0.008 | 0.015 | 5.99E-01 | 172996 | glgc_gwas | intron_variant |
| IGAP_2019 | PCSK9 | rs114162366 | 1 | 55525944 | A | G | 0.027 | 0.016 | 0.032 | 6.19E-01 | 0.014 | 0.056 | 7.98E-01 | 21555 | magnetic | intron_variant |
| IGAP_2019 | PCSK9 | rs11591147 | 1 | 55505647 | T | G | 0.015 | -0.475 | 0.011 | 1.00E-200 | 0.030 | 0.078 | 6.95E-01 | 265213 | glgc_exome | missense_variant |
| IGAP_2019 | PCSK9 | rs11808052 | 1 | 55521352 | T | C | 0.028 | -0.049 | 0.032 | 1.38E-01 | 0.052 | 0.036 | 1.52E-01 | 21555 | magnetic | intron_variant |
| IGAP_2019 | PCSK9 | rs12136600 | 1 | 55521195 | T | C | 0.162 | -0.006 | 0.015 | 6.92E-01 | 0.027 | 0.029 | 3.47E-01 | 19269 | magnetic | intron_variant |
| IGAP_2019 | PCSK9 | rs137886411 | 1 | 55525722 | A | G | 0.005 | 0.190 | 0.097 | 5.39E-02 | 0.031 | 0.083 | 7.10E-01 | 18604 | magnetic | intron_variant |
| IGAP_2019 | PCSK9 | rs146741639 | 1 | 55515334 | T | G | 0.005 | -0.029 | 0.078 | 7.14E-01 | -0.099 | 0.091 | 2.77E-01 | 21553 | magnetic | intron_variant |
| IGAP_2019 | PCSK9 | rs150119739 | 1 | 55520938 | A | G | 0.026 | 0.118 | 0.039 | 3.16E-03 | 0.005 | 0.041 | 9.05E-01 | 19270 | magnetic | intron_variant |
| IGAP_2019 | PCSK9 | rs2479409 | 1 | 55504650 | A | G | 0.657 | -0.047 | 0.003 | 1.00E-200 | -0.012 | 0.016 | 4.47E-01 | 295826 | glgc_exome | upstream_gene_variant |
| IGAP_2019 | PCSK9 | rs2483205 | 1 | 55518316 | C | T | 0.532 | 0.051 | 0.005 | 4.74E-20 | -0.046 | 0.017 | 5.80E-03 | 77862 | glgc_gwas | splice_region_variant |
| IGAP_2019 | PCSK9 | rs2495477 | 1 | 55518467 | T | C | 0.600 | 0.064 | 0.005 | 7.29E-30 | -0.060 | 0.016 | 2.07E-04 | 80151 | glgc_gwas | splice_region_variant |
| IGAP_2019 | PCSK9 | rs2495478 | 1 | 55512995 | A | G | 0.055 | 0.003 | 0.009 | 4.04E-01 | 0.072 | 0.033 | 2.93E-02 | 172338 | glgc_gwas | intron_variant |
| IGAP_2019 | PCSK9 | rs28385708 | 1 | 55513043 | C | T | 0.954 | 0.009 | 0.014 | 4.07E-01 | -0.046 | 0.044 | 3.03E-01 | 77883 | glgc_gwas | intron_variant |
| IGAP_2019 | PCSK9 | rs41294821 | 1 | 55513183 | C | T | 0.970 | 0.030 | 0.020 | 2.86E-01 | -0.089 | 0.046 | 5.48E-02 | 82060 | glgc_gwas | intron_variant |
| IGAP_2019 | PCSK9 | rs45508296 | 1 | 55520547 | G | A | 0.030 | -0.030 | 0.032 | 3.56E-01 | 0.011 | 0.055 | 8.38E-01 | 21554 | magnetic | intron_variant |
| IGAP_2019 | PCSK9 | rs45576433 | 1 | 55517301 | G | A | 0.119 | -0.007 | 0.017 | 6.77E-01 | -0.020 | 0.025 | 4.36E-01 | 19271 | magnetic | non_coding_transcript_exon_variant |
| IGAP_2019 | PCSK9 | rs4927193 | 1 | 55509872 | T | C | 0.869 | 0.035 | 0.006 | 4.27E-11 | 0.009 | 0.021 | 6.57E-01 | 173009 | glgc_gwas | intron_variant |
| IGAP_2019 | PCSK9 | rs499718 | 1 | 55512549 | C | T | 0.826 | 0.036 | 0.007 | 1.13E-06 | -0.016 | 0.019 | 3.83E-01 | 89888 | glgc_gwas | intron_variant |
| IGAP_2019 | PCSK9 | rs505151 | 1 | 55529187 | A | G | 0.949 | -0.090 | 0.006 | 1.00E-200 | -0.041 | 0.038 | 2.81E-01 | 290057 | glgc_exome | missense_variant |
| IGAP_2019 | PCSK9 | rs557435 | 1 | 55520864 | G | A | 0.792 | 0.062 | 0.007 | 6.27E-20 | -0.053 | 0.018 | 3.63E-03 | 106382 | glgc_gwas | intron_variant |
| IGAP_2019 | PCSK9 | rs572512 | 1 | 55517344 | T | C | 0.346 | 0.048 | 0.005 | 5.31E-26 | -0.037 | 0.017 | 2.99E-02 | 150564 | glgc_gwas | non_coding_transcript_exon_variant |
| IGAP_2019 | PCSK9 | rs585131 | 1 | 55524116 | T | C | 0.815 | 0.064 | 0.005 | 2.70E-35 | -0.062 | 0.019 | 9.84E-04 | 167769 | glgc_gwas | intron_variant |
| IGAP_2019 | PCSK9 | rs625619 | 1 | 55518166 | A | G | 0.544 | 0.043 | 0.005 | 7.25E-14 | -0.058 | 0.017 | 4.31E-04 | 77853 | glgc_gwas | intron_variant |
| IGAP_2019 | PCSK9 | rs630431 | 1 | 55527323 | A | G | 0.691 | 0.035 | 0.004 | 7.73E-17 | -0.062 | 0.016 | 7.98E-05 | 166988 | glgc_gwas | intron_variant |
| IGAP_2019 | PCSK9 | rs639750 | 1 | 55519015 | T | G | 0.616 | 0.067 | 0.011 | 9.99E-10 | -0.055 | 0.016 | 8.55E-04 | 21553 | magnetic | intron_variant |
| IGAP_2019 | PCSK9 | rs6681159 | 1 | 55507882 | T | C | 0.790 | 0.065 | 0.013 | 1.08E-06 | -0.008 | 0.018 | 6.32E-01 | 21554 | magnetic | intron_variant |
| IGAP_2019 | PCSK9 | rs74700387 | 1 | 55510015 | C | T | 0.978 | 0.033 | 0.020 | 7.60E-02 | 0.002 | 0.057 | 9.69E-01 | 71313 | glgc_gwas | intron_variant |
| IGAP_2019 | PCSK9 | rs7525503 | 1 | 55522558 | T | G | 0.028 | 0.040 | 0.034 | 2.44E-01 | 0.040 | 0.060 | 5.07E-01 | 19270 | magnetic | intron_variant |
| IGAP_2019 | PCSK9 | rs7552841 | 1 | 55518752 | T | C | 0.365 | 0.037 | 0.004 | 5.40E-15 | -0.044 | 0.017 | 1.00E-02 | 140234 | glgc_gwas | intron_variant |
| IGAP_2019 | PCSK9 | rs79844613 | 1 | 55522415 | A | G | 0.019 | -0.076 | 0.044 | 8.82E-02 | 0.050 | 0.046 | 2.76E-01 | 19270 | magnetic | intron_variant |
| PGC | APOB | rs10199768 | 2 | 21244000 | T | G | 0.385 | 0.097 | 0.004 | 1.00E-200 | -0.006 | 0.038 | 8.74E-01 | 170875 | glgc_gwas | intron_variant |
| PGC | APOB | rs1042023 | 2 | 21229446 | C | G | 0.009 | 0.118 | 0.014 | 2.54E-17 | -0.084 | 0.163 | 6.04E-01 | 276999 | glgc_exome | missense_variant |
| PGC | APOB | rs1042031 | 2 | 21225753 | T | C | 0.172 | -0.045 | 0.004 | 3.04E-37 | 0.048 | 0.050 | 3.35E-01 | 295826 | glgc_exome | stop_gained |
| PGC | APOB | rs11676704 | 2 | 21244358 | T | G | 0.844 | 0.001 | 0.005 | 6.16E-01 | 0.008 | 0.049 | 8.68E-01 | 173023 | glgc_gwas | intron_variant |
| PGC | APOB | rs12691202 | 2 | 21249716 | C | T | 0.949 | 0.097 | 0.011 | 8.22E-19 | -0.077 | 0.110 | 4.84E-01 | 109096 | glgc_gwas | missense_variant |
| PGC | APOB | rs12713771 | 2 | 21235565 | T | C | 0.018 | 0.022 | 0.037 | 5.53E-01 | 0.047 | 0.192 | 8.08E-01 | 21554 | magnetic | intron_variant |
| PGC | APOB | rs12713870 | 2 | 21239595 | G | A | 0.022 | 0.036 | 0.037 | 3.34E-01 | -0.063 | 0.125 | 6.14E-01 | 21553 | magnetic | intron_variant |
| PGC | APOB | rs12720796 | 2 | 21261998 | C | A | 0.022 | 0.091 | 0.014 | 1.68E-10 | 0.041 | 0.112 | 7.17E-01 | 153714 | glgc_gwas | intron_variant |
| PGC | APOB | rs12720816 | 2 | 21239667 | C | T | 0.329 | 0.090 | 0.011 | 1.02E-16 | 0.020 | 0.040 | 6.08E-01 | 21556 | magnetic | intron_variant |
| PGC | APOB | rs12720842 | 2 | 21257927 | C | T | 0.021 | 0.099 | 0.012 | 1.88E-15 | 0.281 | 0.124 | 2.32E-02 | 161300 | glgc_gwas | intron_variant |
| PGC | APOB | rs1367117 | 2 | 21263900 | A | G | 0.285 | 0.105 | 0.003 | 1.00E-200 | 0.017 | 0.040 | 6.71E-01 | 295826 | glgc_exome | missense_variant |
| PGC | APOB | rs149790267 | 2 | 21224120 | G | A | 0.018 | 0.020 | 0.039 | 6.12E-01 | 0.089 | 0.137 | 5.16E-01 | 21555 | magnetic | upstream_gene_variant |
| PGC | APOB | rs150956240 | 2 | 21238984 | G | A | 0.010 | 0.000 | 0.056 | 9.94E-01 | 0.118 | 0.199 | 5.53E-01 | 21554 | magnetic | intron_variant |
| PGC | APOB | rs1801695 | 2 | 21224853 | C | T | 0.974 | 0.002 | 0.014 | 7.41E-01 | 0.000 | 0.140 | 1.00E+00 | 105182 | glgc_gwas | missense_variant |
| PGC | APOB | rs1801699 | 2 | 21233999 | C | T | 0.018 | 0.075 | 0.010 | 8.56E-14 | -0.065 | 0.159 | 6.80E-01 | 273292 | glgc_exome | missense_variant |
| PGC | APOB | rs1801700 | 2 | 21245813 | A | G | 0.042 | 0.018 | 0.008 | 9.56E-02 | 0.163 | 0.100 | 1.01E-01 | 168112 | glgc_gwas | missense_variant |
| PGC | APOB | rs1801701 | 2 | 21228827 | T | C | 0.081 | 0.046 | 0.005 | 4.87E-21 | 0.068 | 0.067 | 3.07E-01 | 293853 | glgc_exome | missense_variant |
| PGC | APOB | rs1801702 | 2 | 21225485 | G | C | 0.028 | -0.091 | 0.008 | 3.66E-30 | 0.102 | 0.126 | 4.19E-01 | 295826 | glgc_exome | missense_variant |
| PGC | APOB | rs184507838 | 2 | 21255764 | T | C | 0.031 | -0.099 | 0.029 | 6.12E-04 | -0.218 | 0.201 | 2.80E-01 | 21553 | magnetic | intron_variant |
| PGC | APOB | rs2678379 | 2 | 21226560 | G | A | 0.768 | 0.058 | 0.004 | 1.65E-38 | 0.068 | 0.047 | 1.51E-01 | 173015 | glgc_gwas | intron_variant |
| PGC | APOB | rs2854725 | 2 | 21237786 | T | G | 0.901 | 0.064 | 0.007 | 4.99E-19 | -0.067 | 0.079 | 3.92E-01 | 172961 | glgc_gwas | intron_variant |
| PGC | APOB | rs3791981 | 2 | 21245367 | A | G | 0.880 | 0.094 | 0.007 | 1.00E-200 | -0.063 | 0.075 | 3.97E-01 | 161484 | glgc_gwas | intron_variant |
| PGC | APOB | rs512535 | 2 | 21267782 | T | C | 0.484 | 0.031 | 0.004 | 2.32E-15 | 0.040 | 0.038 | 2.93E-01 | 172987 | glgc_gwas | upstream_gene_variant |
| PGC | APOB | rs531819 | 2 | 21263639 | G | T | 0.809 | 0.134 | 0.005 | 1.00E-200 | -0.059 | 0.059 | 3.14E-01 | 173033 | glgc_gwas | intron_variant |
| PGC | APOB | rs533617 | 2 | 21233972 | C | T | 0.039 | -0.127 | 0.007 | 1.00E-200 | -0.103 | 0.097 | 2.89E-01 | 291354 | glgc_exome | missense_variant |
| PGC | APOB | rs679899 | 2 | 21250914 | A | G | 0.478 | -0.045 | 0.003 | 1.00E-200 | -0.008 | 0.038 | 8.35E-01 | 295826 | glgc_exome | missense_variant |
| PGC | HMGCR | rs10045497 | 5 | 74636484 | A | C | 0.408 | 0.077 | 0.005 | 1.00E-200 | 0.013 | 0.039 | 7.46E-01 | 89888 | glgc_gwas | intron_variant |
| PGC | HMGCR | rs10474434 | 5 | 74644681 | T | G | 0.252 | 0.063 | 0.006 | 4.11E-22 | -0.043 | 0.045 | 3.47E-01 | 83136 | glgc_gwas | intron_variant |
| PGC | HMGCR | rs10474435 | 5 | 74657280 | C | T | 0.009 | 0.054 | 0.015 | 2.36E-03 | 0.115 | 0.153 | 4.52E-01 | 150792 | glgc_gwas | 3_prime_UTR_variant |
| PGC | HMGCR | rs10515198 | 5 | 74641560 | A | G | 0.103 | 0.060 | 0.006 | 5.99E-22 | 0.149 | 0.063 | 1.87E-02 | 173012 | glgc_gwas | intron_variant |
| PGC | HMGCR | rs142550951 | 5 | 74649285 | T | C | 0.051 | 0.040 | 0.023 | 8.86E-02 | -0.247 | 0.110 | 2.46E-02 | 21554 | magnetic | intron_variant |
| PGC | HMGCR | rs142563098 | 5 | 74633014 | C | T | 0.006 | 0.022 | 0.107 | 8.43E-01 | 0.049 | 0.179 | 7.83E-01 | 14004 | magnetic | 5_prime_UTR_variant |
| PGC | HMGCR | rs17238540 | 5 | 74655498 | T | G | 0.979 | 0.024 | 0.016 | 1.58E-01 | -0.011 | 0.116 | 9.23E-01 | 84983 | glgc_gwas | non_coding_transcript_exon_variant |
| PGC | HMGCR | rs17238596 | 5 | 74652059 | T | C | 0.047 | -0.081 | 0.028 | 5.08E-03 | 0.162 | 0.128 | 2.07E-01 | 19269 | magnetic | intron_variant |
| PGC | HMGCR | rs17244792 | 5 | 74640625 | A | G | 0.023 | -0.041 | 0.040 | 3.09E-01 | 0.342 | 0.140 | 1.43E-02 | 19270 | magnetic | intron_variant |
| PGC | HMGCR | rs17648121 | 5 | 74650106 | T | C | 0.045 | 0.054 | 0.026 | 3.98E-02 | -0.038 | 0.105 | 7.16E-01 | 19266 | magnetic | intron_variant |
| PGC | HMGCR | rs2303152 | 5 | 74641707 | A | G | 0.120 | 0.042 | 0.006 | 1.04E-09 | -0.065 | 0.068 | 3.36E-01 | 160116 | glgc_gwas | intron_variant |
| PGC | HMGCR | rs5908 | 5 | 74652199 | G | A | 0.018 | -0.036 | 0.010 | 2.32E-04 | 0.053 | 0.201 | 7.94E-01 | 290263 | glgc_exome | missense_variant |
| PGC | HMGCR | rs76475757 | 5 | 74637213 | C | T | 0.061 | 0.056 | 0.010 | 4.88E-07 | 0.105 | 0.076 | 1.69E-01 | 83142 | glgc_gwas | intron_variant |
| PGC | HMGCR | rs7717396 | 5 | 74654779 | T | A | 0.054 | 0.018 | 0.008 | 1.11E-02 | -0.048 | 0.081 | 5.56E-01 | 170054 | glgc_gwas | intron_variant |
| PGC | NPC1L1 | rs10264715 | 7 | 44555406 | A | G | 0.199 | 0.022 | 0.004 | 1.65E-06 | 0.049 | 0.043 | 2.52E-01 | 173026 | glgc_gwas | synonymous_variant |
| PGC | NPC1L1 | rs113223818 | 7 | 44577569 | A | G | 0.025 | 0.003 | 0.020 | 7.91E-01 | -0.074 | 0.144 | 6.09E-01 | 54559 | glgc_gwas | intron_variant |
| PGC | NPC1L1 | rs117448375 | 7 | 44566246 | T | C | 0.007 | -0.089 | 0.075 | 2.43E-01 | 0.083 | 0.142 | 5.58E-01 | 21556 | magnetic | intron_variant |
| PGC | NPC1L1 | rs117788436 | 7 | 44559271 | C | G | 0.014 | 0.004 | 0.054 | 9.45E-01 | 0.159 | 0.187 | 3.95E-01 | 19264 | magnetic | intron_variant |
| PGC | NPC1L1 | rs139264665 | 7 | 44564912 | G | A | 0.019 | 0.059 | 0.046 | 2.06E-01 | 0.144 | 0.119 | 2.27E-01 | 19269 | magnetic | intron_variant |
| PGC | NPC1L1 | rs139912571 | 7 | 44565670 | A | C | 0.019 | 0.040 | 0.042 | 3.52E-01 | -0.014 | 0.112 | 9.01E-01 | 21553 | magnetic | intron_variant |
| PGC | NPC1L1 | rs142301466 | 7 | 44558216 | T | C | 0.010 | -0.058 | 0.058 | 3.17E-01 | -0.452 | 0.243 | 6.27E-02 | 19267 | magnetic | synonymous_variant |
| PGC | NPC1L1 | rs142708379 | 7 | 44566281 | G | C | 0.085 | 0.022 | 0.018 | 2.32E-01 | -0.037 | 0.083 | 6.59E-01 | 21555 | magnetic | intron_variant |
| PGC | NPC1L1 | rs17655652 | 7 | 44580991 | T | C | 0.711 | 0.028 | 0.004 | 2.18E-10 | -0.008 | 0.043 | 8.50E-01 | 162152 | glgc_gwas | upstream_gene_variant |
| PGC | NPC1L1 | rs186548547 | 7 | 44554349 | G | C | 0.021 | 0.046 | 0.037 | 2.14E-01 | -0.050 | 0.109 | 6.47E-01 | 21553 | magnetic | intron_variant |
| PGC | NPC1L1 | rs2072183 | 7 | 44579180 | C | G | 0.237 | 0.039 | 0.005 | 7.12E-16 | 0.048 | 0.045 | 2.90E-01 | 169790 | glgc_gwas | synonymous_variant |
| PGC | NPC1L1 | rs2073548 | 7 | 44581620 | G | A | 0.061 | 0.014 | 0.013 | 4.25E-01 | 0.072 | 0.091 | 4.32E-01 | 68864 | glgc_gwas | upstream_gene_variant |
| PGC | NPC1L1 | rs217406 | 7 | 44573761 | G | C | 0.164 | 0.039 | 0.005 | 4.09E-14 | 0.022 | 0.051 | 6.69E-01 | 169605 | glgc_gwas | intron_variant |
| PGC | NPC1L1 | rs217426 | 7 | 44558859 | A | C | 0.974 | 0.005 | 0.011 | 6.64E-01 | -0.089 | 0.109 | 4.17E-01 | 164788 | glgc_gwas | intron_variant |
| PGC | NPC1L1 | rs217432 | 7 | 44553826 | G | A | 0.195 | 0.022 | 0.006 | 5.84E-04 | 0.104 | 0.043 | 1.58E-02 | 89888 | glgc_gwas | intron_variant |
| PGC | NPC1L1 | rs35349497 | 7 | 44561635 | C | T | 0.905 | 0.018 | 0.009 | 5.90E-02 | -0.010 | 0.088 | 9.07E-01 | 82303 | glgc_gwas | intron_variant |
| PGC | NPC1L1 | rs41279627 | 7 | 44571628 | C | T | 0.954 | 0.026 | 0.013 | 8.03E-02 | 0.054 | 0.119 | 6.51E-01 | 83146 | glgc_gwas | intron_variant |
| PGC | NPC1L1 | rs41279630 | 7 | 44573803 | A | G | 0.012 | -0.153 | 0.052 | 4.12E-03 | 0.517 | 0.216 | 1.66E-02 | 19270 | magnetic | intron_variant |
| PGC | NPC1L1 | rs4720470 | 7 | 44561884 | C | T | 0.933 | 0.002 | 0.010 | 9.44E-01 | -0.058 | 0.106 | 5.85E-01 | 117734 | glgc_gwas | intron_variant |
| PGC | NPC1L1 | rs52815063 | 7 | 44555699 | T | A | 0.010 | 0.017 | 0.019 | 3.70E-01 | 0.150 | 0.203 | 4.62E-01 | 129808 | glgc_exome | missense_variant |
| PGC | NPC1L1 | rs56243746 | 7 | 44581150 | G | A | 0.021 | 0.018 | 0.016 | 2.54E-01 | -0.115 | 0.184 | 5.33E-01 | 72546 | glgc_gwas | upstream_gene_variant |
| PGC | NPC1L1 | rs62459147 | 7 | 44566917 | T | C | 0.052 | -0.043 | 0.025 | 9.05E-02 | 0.078 | 0.136 | 5.66E-01 | 19267 | magnetic | intron_variant |
| PGC | NPC1L1 | rs73107472 | 7 | 44575664 | T | G | 0.013 | 0.009 | 0.022 | 8.73E-01 | 0.044 | 0.155 | 7.76E-01 | 77803 | glgc_gwas | intron_variant |
| PGC | NPC1L1 | rs77826622 | 7 | 44572346 | C | T | 0.017 | 0.063 | 0.045 | 1.74E-01 | 0.058 | 0.143 | 6.86E-01 | 21554 | magnetic | intron_variant |
| PGC | NPC1L1 | rs7808295 | 7 | 44572699 | T | C | 0.028 | -0.024 | 0.035 | 4.96E-01 | -0.023 | 0.108 | 8.31E-01 | 21554 | magnetic | intron_variant |
| PGC | PCSK9 | rs11206514 | 1 | 55516004 | A | C | 0.611 | 0.051 | 0.004 | 9.95E-33 | -0.072 | 0.040 | 6.88E-02 | 172996 | glgc_gwas | intron_variant |
| PGC | PCSK9 | rs114162366 | 1 | 55525944 | A | G | 0.027 | 0.016 | 0.032 | 6.19E-01 | -0.011 | 0.104 | 9.15E-01 | 21555 | magnetic | intron_variant |
| PGC | PCSK9 | rs11591147 | 1 | 55505647 | T | G | 0.015 | -0.475 | 0.011 | 1.00E-200 | 0.198 | 0.182 | 2.76E-01 | 265213 | glgc_exome | missense_variant |
| PGC | PCSK9 | rs11808052 | 1 | 55521352 | T | C | 0.028 | -0.049 | 0.032 | 1.38E-01 | 0.063 | 0.093 | 4.96E-01 | 21555 | magnetic | intron_variant |
| PGC | PCSK9 | rs12136600 | 1 | 55521195 | T | C | 0.162 | -0.006 | 0.015 | 6.92E-01 | 0.085 | 0.067 | 2.05E-01 | 19269 | magnetic | intron_variant |
| PGC | PCSK9 | rs146741639 | 1 | 55515334 | T | G | 0.005 | -0.029 | 0.078 | 7.14E-01 | -0.410 | 0.184 | 2.60E-02 | 21553 | magnetic | intron_variant |
| PGC | PCSK9 | rs150119739 | 1 | 55520938 | A | G | 0.026 | 0.118 | 0.039 | 3.16E-03 | -0.040 | 0.104 | 7.03E-01 | 19270 | magnetic | intron_variant |
| PGC | PCSK9 | rs2479409 | 1 | 55504650 | A | G | 0.657 | -0.047 | 0.003 | 1.00E-200 | 0.057 | 0.040 | 1.60E-01 | 295826 | glgc_exome | upstream_gene_variant |
| PGC | PCSK9 | rs2483205 | 1 | 55518316 | C | T | 0.532 | 0.051 | 0.005 | 4.74E-20 | -0.058 | 0.038 | 1.32E-01 | 77862 | glgc_gwas | splice_region_variant |
| PGC | PCSK9 | rs2495477 | 1 | 55518467 | T | C | 0.600 | 0.064 | 0.005 | 7.29E-30 | -0.044 | 0.039 | 2.63E-01 | 80151 | glgc_gwas | splice_region_variant |
| PGC | PCSK9 | rs2495481 | 1 | 55508186 | T | A | 0.055 | 0.008 | 0.009 | 2.39E-01 | -0.092 | 0.090 | 3.07E-01 | 172296 | glgc_gwas | intron_variant |
| PGC | PCSK9 | rs28385708 | 1 | 55513043 | C | T | 0.954 | 0.009 | 0.014 | 4.07E-01 | -0.136 | 0.121 | 2.61E-01 | 77883 | glgc_gwas | intron_variant |
| PGC | PCSK9 | rs41294821 | 1 | 55513183 | C | T | 0.970 | 0.030 | 0.020 | 2.86E-01 | 0.127 | 0.158 | 4.20E-01 | 82060 | glgc_gwas | intron_variant |
| PGC | PCSK9 | rs41297885 | 1 | 55526840 | G | C | 0.038 | -0.016 | 0.026 | 5.56E-01 | 0.019 | 0.099 | 8.50E-01 | 21554 | magnetic | intron_variant |
| PGC | PCSK9 | rs45508296 | 1 | 55520547 | G | A | 0.030 | -0.030 | 0.032 | 3.56E-01 | 0.170 | 0.130 | 1.88E-01 | 21554 | magnetic | intron_variant |
| PGC | PCSK9 | rs45576433 | 1 | 55517301 | G | A | 0.119 | -0.007 | 0.017 | 6.77E-01 | 0.053 | 0.060 | 3.73E-01 | 19271 | magnetic | non_coding_transcript_exon_variant |
| PGC | PCSK9 | rs4927193 | 1 | 55509872 | T | C | 0.869 | 0.035 | 0.006 | 4.27E-11 | -0.043 | 0.056 | 4.43E-01 | 173009 | glgc_gwas | intron_variant |
| PGC | PCSK9 | rs499718 | 1 | 55512549 | C | T | 0.826 | 0.036 | 0.007 | 1.13E-06 | -0.013 | 0.050 | 8.03E-01 | 89888 | glgc_gwas | intron_variant |
| PGC | PCSK9 | rs505151 | 1 | 55529187 | A | G | 0.949 | -0.090 | 0.006 | 1.00E-200 | 0.194 | 0.095 | 4.14E-02 | 290057 | glgc_exome | missense_variant |
| PGC | PCSK9 | rs529787 | 1 | 55513521 | C | G | 0.782 | 0.055 | 0.005 | 8.75E-24 | -0.079 | 0.052 | 1.29E-01 | 161969 | glgc_gwas | intron_variant |
| PGC | PCSK9 | rs557435 | 1 | 55520864 | G | A | 0.792 | 0.062 | 0.007 | 6.27E-20 | -0.050 | 0.047 | 2.92E-01 | 106382 | glgc_gwas | intron_variant |
| PGC | PCSK9 | rs572512 | 1 | 55517344 | T | C | 0.346 | 0.048 | 0.005 | 5.31E-26 | 0.011 | 0.041 | 7.85E-01 | 150564 | glgc_gwas | non_coding_transcript_exon_variant |
| PGC | PCSK9 | rs585131 | 1 | 55524116 | T | C | 0.815 | 0.064 | 0.005 | 2.70E-35 | -0.039 | 0.051 | 4.46E-01 | 167769 | glgc_gwas | intron_variant |
| PGC | PCSK9 | rs624612 | 1 | 55517883 | G | C | 0.415 | 0.059 | 0.012 | 5.02E-07 | -0.019 | 0.041 | 6.36E-01 | 19269 | magnetic | non_coding_transcript_exon_variant |
| PGC | PCSK9 | rs625619 | 1 | 55518166 | A | G | 0.544 | 0.043 | 0.005 | 7.25E-14 | -0.014 | 0.041 | 7.25E-01 | 77853 | glgc_gwas | intron_variant |
| PGC | PCSK9 | rs630431 | 1 | 55527323 | A | G | 0.691 | 0.035 | 0.004 | 7.73E-17 | -0.014 | 0.041 | 7.41E-01 | 166988 | glgc_gwas | intron_variant |
| PGC | PCSK9 | rs639750 | 1 | 55519015 | T | G | 0.616 | 0.067 | 0.011 | 9.99E-10 | -0.052 | 0.040 | 1.95E-01 | 21553 | magnetic | intron_variant |
| PGC | PCSK9 | rs74700387 | 1 | 55510015 | C | T | 0.978 | 0.033 | 0.020 | 7.60E-02 | -0.113 | 0.138 | 4.15E-01 | 71313 | glgc_gwas | intron_variant |
| PGC | PCSK9 | rs7525503 | 1 | 55522558 | T | G | 0.028 | 0.040 | 0.034 | 2.44E-01 | -0.261 | 0.134 | 5.20E-02 | 19270 | magnetic | intron_variant |
| PGC | PCSK9 | rs7552841 | 1 | 55518752 | T | C | 0.365 | 0.037 | 0.004 | 5.40E-15 | -0.017 | 0.045 | 7.11E-01 | 140234 | glgc_gwas | intron_variant |
| PGC | PCSK9 | rs79844613 | 1 | 55522415 | A | G | 0.019 | -0.076 | 0.044 | 8.82E-02 | -0.064 | 0.133 | 6.31E-01 | 19270 | magnetic | intron_variant |

*Abbreviations: Chr – chromosome; EAF - effect allele frequency; glgc_exome - Global Lipid Genetics Consortium exome-wide study; glgc_gwas - Global Lipid Genetics Consortium genome-wide association study; IGAP - International Genomics of Alzheimer's disease project; PGC - Psychiatric Genomics Consortium; SNP - single nucleotide polymorphism*

Foot notes:

1) SNPs were selected from within gene start and stop coordinates ±1kb

2) SNP positions are from human reference genome build GRChr37

3) P values for association with LDL-C were recorded with a lower limit of 1.00E-200

4) Functional annotations for SNPs were derived from Ensembl Variant Effect Predictor

# Supplementary table 3: information on alternate gene-specific variant sets used to assess the effects of lipid-lowering drug targets on AD risk in IVW MR models with uncorrelated variants

| **Study** | **Gene** | **SNP** | **Chr** | **Position** | **Effect allele** | **Alt allele** | **EAF exposure** | **Beta exposure** | **SE exposure** | **P exposure** | **Beta outcome** | **SE outcome** | **P outcome** | **Sample size exposure** | **Study exposure** | **Functional**  **consequence** |
| --- | --- | --- | --- | --- | --- | --- | --- | --- | --- | --- | --- | --- | --- | --- | --- | --- |
| IGAP_2019 | HMGCR | rs10062361 | 5 | 74565153 | T | C | 0.235 | 0.069 | 0.004 | 1.00E-200 | 0.009 | 0.017 | 6.04E-01 | 173022 | glgc_gwas | intergenic_variant |
| IGAP_2019 | HMGCR | rs3761739 | 5 | 74631501 | T | C | 0.157 | 0.046 | 0.005 | 7.16E-20 | -0.053 | 0.020 | 7.14E-03 | 173025 | glgc_gwas | intron_variant |
| IGAP_2019 | HMGCR | rs3857388 | 5 | 74620377 | C | T | 0.128 | 0.042 | 0.006 | 2.20E-11 | 0.021 | 0.022 | 3.49E-01 | 172939 | glgc_gwas | intron_variant |
| IGAP_2019 | HMGCR | rs6453133 | 5 | 74692776 | G | A | 0.309 | 0.050 | 0.004 | 1.08E-32 | 0.023 | 0.016 | 1.40E-01 | 173030 | glgc_gwas | intron_variant |
| IGAP_2019 | HMGCR | rs7711235 | 5 | 74540397 | G | A | 0.268 | 0.038 | 0.006 | 5.00E-10 | 0.002 | 0.017 | 8.99E-01 | 89724 | glgc_gwas | intergenic_variant |
| IGAP_2019 | PCSK9 | rs11206510 | 1 | 55496039 | C | T | 0.167 | -0.070 | 0.004 | 1.00E-200 | 0.004 | 0.018 | 8.15E-01 | 294565 | glgc_exome | intergenic_variant |
| IGAP_2019 | PCSK9 | rs11206514 | 1 | 55516004 | A | C | 0.611 | 0.051 | 0.004 | 9.95E-33 | -0.008 | 0.015 | 5.99E-01 | 172996 | glgc_gwas | intron_variant |
| IGAP_2019 | PCSK9 | rs11580527 | 1 | 55411580 | A | G | 0.036 | -0.366 | 0.030 | 1.13E-33 | -0.012 | 0.095 | 8.99E-01 | 19265 | magnetic | intergenic_variant |
| IGAP_2019 | PCSK9 | rs11583974 | 1 | 55551718 | A | G | 0.030 | 0.065 | 0.012 | 3.95E-09 | -0.063 | 0.051 | 2.12E-01 | 99955 | glgc_gwas | splice_region_variant |
| IGAP_2019 | PCSK9 | rs11591147 | 1 | 55505647 | T | G | 0.015 | -0.475 | 0.011 | 1.00E-200 | 0.030 | 0.078 | 6.95E-01 | 265213 | glgc_exome | missense_variant |
| IGAP_2019 | PCSK9 | rs12043403 | 1 | 55431933 | C | T | 0.112 | -0.139 | 0.018 | 2.37E-14 | -0.024 | 0.029 | 4.12E-01 | 19270 | magnetic | regulatory_region_variant |
| IGAP_2019 | PCSK9 | rs2479394 | 1 | 55486064 | G | A | 0.285 | 0.039 | 0.004 | 1.58E-19 | 0.005 | 0.016 | 7.68E-01 | 172953 | glgc_gwas | intergenic_variant |
| IGAP_2019 | PCSK9 | rs2495477 | 1 | 55518467 | T | C | 0.600 | 0.064 | 0.005 | 7.29E-30 | -0.060 | 0.016 | 2.07E-04 | 80151 | glgc_gwas | splice_region_variant |
| IGAP_2019 | PCSK9 | rs505151 | 1 | 55529187 | A | G | 0.949 | -0.090 | 0.006 | 1.00E-200 | -0.041 | 0.038 | 2.81E-01 | 290057 | glgc_exome | missense_variant |
| IGAP_2019 | PCSK9 | rs572512 | 1 | 55517344 | T | C | 0.346 | 0.048 | 0.005 | 5.31E-26 | -0.037 | 0.017 | 2.99E-02 | 150564 | glgc_gwas | non_coding_transcript_exon_variant |
| IGAP_2019 | PCSK9 | rs585131 | 1 | 55524116 | T | C | 0.815 | 0.064 | 0.005 | 2.70E-35 | -0.062 | 0.019 | 9.84E-04 | 167769 | glgc_gwas | intron_variant |
| IGAP_2019 | PCSK9 | rs6663252 | 1 | 55630151 | C | T | 0.211 | -0.085 | 0.012 | 5.83E-12 | 0.043 | 0.021 | 4.05E-02 | 21553 | magnetic | intron_variant |
| IGAP_2019 | PCSK9 | rs7523242 | 1 | 55498949 | T | C | 0.162 | 0.069 | 0.005 | 1.00E-200 | -0.001 | 0.018 | 9.79E-01 | 172980 | glgc_gwas | intergenic_variant |
| IGAP_2019 | NPC1L1 | rs2073547 | 7 | 44582331 | G | A | 0.194 | 0.049 | 0.005 | 1.92E-21 | -0.031 | 0.019 | 9.93E-02 | 169889 | glgc_gwas | upstream_gene_variant |
| IGAP_2019 | NPC1L1 | rs217386 | 7 | 44600695 | G | A | 0.592 | 0.036 | 0.004 | 1.20E-19 | -0.007 | 0.015 | 6.56E-01 | 173021 | glgc_gwas | downstream_gene_variant |
| IGAP_2019 | APOB | rs10164442 | 2 | 21205563 | G | A | 0.321 | 0.029 | 0.004 | 7.78E-13 | 0.015 | 0.015 | 3.14E-01 | 173004 | glgc_gwas | intergenic_variant |
| IGAP_2019 | APOB | rs10198175 | 2 | 21133883 | A | G | 0.084 | 0.077 | 0.006 | 1.45E-32 | -0.020 | 0.025 | 4.26E-01 | 172977 | glgc_gwas | intergenic_variant |
| IGAP_2019 | APOB | rs1042031 | 2 | 21225753 | T | C | 0.172 | -0.045 | 0.004 | 3.04E-37 | 0.030 | 0.018 | 1.04E-01 | 295826 | glgc_exome | stop_gained |
| IGAP_2019 | APOB | rs113588790 | 2 | 21364562 | T | C | 0.024 | 0.090 | 0.014 | 3.93E-09 | 0.003 | 0.042 | 9.46E-01 | 82994 | glgc_gwas | missense_variant |
| IGAP_2019 | APOB | rs12720796 | 2 | 21261998 | C | A | 0.022 | 0.091 | 0.014 | 1.68E-10 | -0.003 | 0.061 | 9.57E-01 | 153714 | glgc_gwas | intron_variant |
| IGAP_2019 | APOB | rs13392272 | 2 | 21217490 | T | C | 0.413 | 0.087 | 0.004 | 1.00E-200 | -0.004 | 0.014 | 8.08E-01 | 172951 | glgc_gwas | downstream_gene_variant |
| IGAP_2019 | APOB | rs17398765 | 2 | 21270751 | G | A | 0.069 | 0.092 | 0.008 | 3.54E-32 | 0.038 | 0.032 | 2.38E-01 | 168107 | glgc_gwas | upstream_gene_variant |
| IGAP_2019 | APOB | rs1801701 | 2 | 21228827 | T | C | 0.081 | 0.046 | 0.005 | 4.87E-21 | -0.017 | 0.024 | 4.91E-01 | 293853 | glgc_exome | missense_variant |
| IGAP_2019 | APOB | rs3791980 | 2 | 21245329 | T | G | 0.703 | 0.043 | 0.004 | 9.74E-21 | 0.029 | 0.016 | 6.67E-02 | 144612 | glgc_gwas | intron_variant |
| IGAP_2019 | APOB | rs3791981 | 2 | 21245367 | A | G | 0.880 | 0.094 | 0.007 | 1.00E-200 | -0.043 | 0.024 | 7.62E-02 | 161484 | glgc_gwas | intron_variant |
| IGAP_2019 | APOB | rs4341893 | 2 | 21135577 | A | G | 0.309 | 0.060 | 0.004 | 1.00E-200 | -0.010 | 0.015 | 5.03E-01 | 170089 | glgc_gwas | intergenic_variant |
| IGAP_2019 | APOB | rs481069 | 2 | 21281856 | A | G | 0.823 | 0.135 | 0.007 | 1.00E-200 | -0.021 | 0.020 | 3.09E-01 | 82984 | glgc_gwas | intergenic_variant |
| IGAP_2019 | APOB | rs62120800 | 2 | 21126936 | A | G | 0.040 | -0.191 | 0.026 | 3.40E-13 | 0.070 | 0.070 | 3.19E-01 | 21553 | magnetic | intergenic_variant |
| IGAP_2019 | APOB | rs6547409 | 2 | 21190209 | C | T | 0.941 | 0.123 | 0.009 | 1.00E-200 | 0.015 | 0.036 | 6.79E-01 | 167103 | glgc_gwas | intergenic_variant |
| IGAP_2019 | APOB | rs6548010 | 2 | 21277922 | G | A | 0.313 | 0.106 | 0.004 | 1.00E-200 | 0.011 | 0.015 | 4.89E-01 | 173033 | glgc_gwas | intergenic_variant |
| IGAP_2019 | APOB | rs6756743 | 2 | 21301892 | T | C | 0.042 | 0.055 | 0.009 | 4.97E-09 | 0.072 | 0.033 | 3.09E-02 | 163843 | glgc_gwas | intergenic_variant |
| IGAP_2019 | APOB | rs7567653 | 2 | 21276962 | G | A | 0.963 | 0.115 | 0.011 | 3.37E-26 | 0.007 | 0.040 | 8.70E-01 | 161178 | glgc_gwas | intergenic_variant |
| PGC | HMGCR | rs10062361 | 5 | 74565153 | T | C | 0.235 | 0.069 | 0.004 | 1.00E-200 | 0.060 | 0.045 | 1.83E-01 | 173022 | glgc_gwas | intergenic_variant |
| PGC | HMGCR | rs3761739 | 5 | 74631501 | T | C | 0.157 | 0.046 | 0.005 | 7.16E-20 | 0.078 | 0.052 | 1.32E-01 | 173025 | glgc_gwas | intron_variant |
| PGC | HMGCR | rs3857388 | 5 | 74620377 | C | T | 0.128 | 0.042 | 0.006 | 2.20E-11 | -0.051 | 0.064 | 4.30E-01 | 172939 | glgc_gwas | intron_variant |
| PGC | HMGCR | rs6453133 | 5 | 74692776 | G | A | 0.309 | 0.050 | 0.004 | 1.08E-32 | -0.078 | 0.042 | 6.49E-02 | 173030 | glgc_gwas | intron_variant |
| PGC | HMGCR | rs7711235 | 5 | 74540397 | G | A | 0.268 | 0.038 | 0.006 | 5.00E-10 | -0.006 | 0.047 | 9.06E-01 | 89724 | glgc_gwas | intergenic_variant |
| PGC | PCSK9 | rs11206510 | 1 | 55496039 | C | T | 0.167 | -0.070 | 0.004 | 1.00E-200 | 0.051 | 0.051 | 3.12E-01 | 294565 | glgc_exome | intergenic_variant |
| PGC | PCSK9 | rs11206514 | 1 | 55516004 | A | C | 0.611 | 0.051 | 0.004 | 9.95E-33 | -0.072 | 0.040 | 6.88E-02 | 172996 | glgc_gwas | intron_variant |
| PGC | PCSK9 | rs11583974 | 1 | 55551718 | A | G | 0.030 | 0.065 | 0.012 | 3.95E-09 | 0.043 | 0.113 | 7.05E-01 | 99955 | glgc_gwas | splice_region_variant |
| PGC | PCSK9 | rs11591147 | 1 | 55505647 | T | G | 0.015 | -0.475 | 0.011 | 1.00E-200 | 0.198 | 0.182 | 2.76E-01 | 265213 | glgc_exome | missense_variant |
| PGC | PCSK9 | rs12043403 | 1 | 55431933 | C | T | 0.112 | -0.139 | 0.018 | 2.37E-14 | 0.027 | 0.073 | 7.15E-01 | 19270 | magnetic | regulatory_region_variant |
| PGC | PCSK9 | rs2479394 | 1 | 55486064 | G | A | 0.285 | 0.039 | 0.004 | 1.58E-19 | -0.016 | 0.042 | 7.03E-01 | 172953 | glgc_gwas | intergenic_variant |
| PGC | PCSK9 | rs2495477 | 1 | 55518467 | T | C | 0.600 | 0.064 | 0.005 | 7.29E-30 | -0.044 | 0.039 | 2.63E-01 | 80151 | glgc_gwas | splice_region_variant |
| PGC | PCSK9 | rs505151 | 1 | 55529187 | A | G | 0.949 | -0.090 | 0.006 | 1.00E-200 | 0.194 | 0.095 | 4.14E-02 | 290057 | glgc_exome | missense_variant |
| PGC | PCSK9 | rs572512 | 1 | 55517344 | T | C | 0.346 | 0.048 | 0.005 | 5.31E-26 | 0.011 | 0.041 | 7.85E-01 | 150564 | glgc_gwas | non_coding_transcript_exon_variant |
| PGC | PCSK9 | rs585131 | 1 | 55524116 | T | C | 0.815 | 0.064 | 0.005 | 2.70E-35 | -0.039 | 0.051 | 4.46E-01 | 167769 | glgc_gwas | intron_variant |
| PGC | PCSK9 | rs6663252 | 1 | 55630151 | C | T | 0.211 | -0.085 | 0.012 | 5.83E-12 | 0.070 | 0.054 | 1.92E-01 | 21553 | magnetic | intron_variant |
| PGC | PCSK9 | rs7523242 | 1 | 55498949 | T | C | 0.162 | 0.069 | 0.005 | 1.00E-200 | -0.051 | 0.053 | 3.40E-01 | 172980 | glgc_gwas | intergenic_variant |
| PGC | NPC1L1 | rs2073547 | 7 | 44582331 | G | A | 0.194 | 0.049 | 0.005 | 1.92E-21 | 0.042 | 0.045 | 3.59E-01 | 169889 | glgc_gwas | upstream_gene_variant |
| PGC | NPC1L1 | rs217386 | 7 | 44600695 | G | A | 0.592 | 0.036 | 0.004 | 1.20E-19 | 0.029 | 0.039 | 4.62E-01 | 173021 | glgc_gwas | downstream_gene_variant |
| PGC | APOB | rs10164442 | 2 | 21205563 | G | A | 0.321 | 0.029 | 0.004 | 7.78E-13 | -0.038 | 0.041 | 3.50E-01 | 173004 | glgc_gwas | intergenic_variant |
| PGC | APOB | rs10198175 | 2 | 21133883 | A | G | 0.084 | 0.077 | 0.006 | 1.45E-32 | -0.007 | 0.069 | 9.17E-01 | 172977 | glgc_gwas | intergenic_variant |
| PGC | APOB | rs1042023 | 2 | 21229446 | C | G | 0.009 | 0.118 | 0.014 | 2.54E-17 | -0.084 | 0.163 | 6.04E-01 | 276999 | glgc_exome | missense_variant |
| PGC | APOB | rs1042031 | 2 | 21225753 | T | C | 0.172 | -0.045 | 0.004 | 3.04E-37 | 0.048 | 0.050 | 3.35E-01 | 295826 | glgc_exome | stop_gained |
| PGC | APOB | rs113588790 | 2 | 21364562 | T | C | 0.024 | 0.090 | 0.014 | 3.93E-09 | 0.088 | 0.110 | 4.23E-01 | 82994 | glgc_gwas | missense_variant |
| PGC | APOB | rs12714264 | 2 | 21265518 | A | T | 0.832 | 0.127 | 0.005 | 1.00E-200 | -0.039 | 0.064 | 5.49E-01 | 172866 | glgc_gwas | intron_variant |
| PGC | APOB | rs13392272 | 2 | 21217490 | T | C | 0.413 | 0.087 | 0.004 | 1.00E-200 | 0.002 | 0.039 | 9.68E-01 | 172951 | glgc_gwas | downstream_gene_variant |
| PGC | APOB | rs17398765 | 2 | 21270751 | G | A | 0.069 | 0.092 | 0.008 | 3.54E-32 | -0.110 | 0.079 | 1.62E-01 | 168107 | glgc_gwas | upstream_gene_variant |
| PGC | APOB | rs1801701 | 2 | 21228827 | T | C | 0.081 | 0.046 | 0.005 | 4.87E-21 | 0.068 | 0.067 | 3.07E-01 | 293853 | glgc_exome | missense_variant |
| PGC | APOB | rs3791980 | 2 | 21245329 | T | G | 0.703 | 0.043 | 0.004 | 9.74E-21 | 0.057 | 0.042 | 1.76E-01 | 144612 | glgc_gwas | intron_variant |
| PGC | APOB | rs3791981 | 2 | 21245367 | A | G | 0.880 | 0.094 | 0.007 | 1.00E-200 | -0.063 | 0.075 | 3.97E-01 | 161484 | glgc_gwas | intron_variant |
| PGC | APOB | rs4341893 | 2 | 21135577 | A | G | 0.309 | 0.060 | 0.004 | 1.00E-200 | 0.039 | 0.040 | 3.31E-01 | 170089 | glgc_gwas | intergenic_variant |
| PGC | APOB | rs62120800 | 2 | 21126936 | A | G | 0.040 | -0.191 | 0.026 | 3.40E-13 | 0.067 | 0.157 | 6.67E-01 | 21553 | magnetic | intergenic_variant |
| PGC | APOB | rs6547409 | 2 | 21190209 | C | T | 0.941 | 0.123 | 0.009 | 1.00E-200 | 0.072 | 0.090 | 4.23E-01 | 167103 | glgc_gwas | intergenic_variant |
| PGC | APOB | rs6548010 | 2 | 21277922 | G | A | 0.313 | 0.106 | 0.004 | 1.00E-200 | 0.021 | 0.039 | 5.86E-01 | 173033 | glgc_gwas | intergenic_variant |
| PGC | APOB | rs6756743 | 2 | 21301892 | T | C | 0.042 | 0.055 | 0.009 | 4.97E-09 | 0.057 | 0.101 | 5.71E-01 | 163843 | glgc_gwas | intergenic_variant |
| PGC | APOB | rs7567653 | 2 | 21276962 | G | A | 0.963 | 0.115 | 0.011 | 3.37E-26 | 0.076 | 0.103 | 4.58E-01 | 161178 | glgc_gwas | intergenic_variant |

*Abbreviations: Chr – chromosome; EAF - effect allele frequency; glgc_exome - Global Lipid Genetics Consortium exome-wide study; glgc_gwas - Global Lipid Genetics Consortium genome-wide association study; IGAP - International Genomics of Alzheimer's disease project; PGC - Psychiatric Genomics Consortium; SNP - single nucleotide polymorphism*

Foot notes:

1) SNPs were selected from within gene start and stop coordinates ±100kb

2) These SNP selections were used for liberal IVW models (with LD clumping at r2<0.2), but also contain the more limited variant sets per gene used in conservative IVW models (clumped with r2<0.01)

3) SNP positions are from human reference genome build GRChr37

4) P values for association with LDL-C were recorded with a lower limit of 1.00E-200

5) Functional annotations for SNPs were derived from Ensembl Variant Effect Predictor

# Supplementary table 4: information on gene-specific variant sets used to assess the effects of lipid-lowering drug targets on cardiometabolic outcomes

| **Outcome** | **Gene** | **SNP** | **Chr** | **Position** | **Effect allele** | **Alt allele** | **EAF exposure** | **Beta exposure** | **SE exposure** | **P exposure** | **Beta outcome** | **SE outcome** | **P outcome** | **Sample size exposure** | **Study exposure** | **Functional**  **consequence** |
| --- | --- | --- | --- | --- | --- | --- | --- | --- | --- | --- | --- | --- | --- | --- | --- | --- |
| CAD | HMGCR | rs10045497 | 5 | 74636484 | A | C | 0.408 | 0.077 | 0.005 | 1.00E-200 | 0.039 | 0.015 | 7.69E-03 | 89888 | glgc_gwas | intron_variant |
| CAD | HMGCR | rs10063134 | 5 | 74649649 | C | T | 0.005 | 0.072 | 0.036 | 1.22E-01 | -0.052 | 0.084 | 5.38E-01 | 50241 | glgc_gwas | intron_variant |
| CAD | HMGCR | rs10474435 | 5 | 74657280 | C | T | 0.009 | 0.054 | 0.015 | 2.36E-03 | -0.012 | 0.061 | 8.40E-01 | 150792 | glgc_gwas | 3_prime_UTR_variant |
| CAD | HMGCR | rs10515198 | 5 | 74641560 | A | G | 0.103 | 0.060 | 0.006 | 5.99E-22 | 0.055 | 0.023 | 1.92E-02 | 173012 | glgc_gwas | intron_variant |
| CAD | HMGCR | rs11742194 | 5 | 74646878 | T | C | 0.103 | 0.059 | 0.006 | 8.13E-22 | 0.054 | 0.023 | 1.98E-02 | 173026 | glgc_gwas | intron_variant |
| CAD | HMGCR | rs12654264 | 5 | 74648603 | T | A | 0.401 | 0.066 | 0.003 | 1.00E-200 | 0.035 | 0.014 | 1.60E-02 | 295826 | glgc_exome | intron_variant |
| CAD | HMGCR | rs2303151 | 5 | 74655451 | T | C | 0.054 | 0.015 | 0.008 | 2.20E-02 | -0.014 | 0.033 | 6.75E-01 | 170080 | glgc_gwas | non_coding_transcript_exon_variant |
| CAD | HMGCR | rs2303152 | 5 | 74641707 | A | G | 0.120 | 0.042 | 0.006 | 1.04E-09 | 0.012 | 0.024 | 6.26E-01 | 160116 | glgc_gwas | intron_variant |
| CAD | HMGCR | rs3761739 | 5 | 74631501 | T | C | 0.157 | 0.046 | 0.005 | 7.16E-20 | 0.028 | 0.021 | 1.75E-01 | 173025 | glgc_gwas | intron_variant |
| CAD | HMGCR | rs3761740 | 5 | 74632133 | A | C | 0.103 | 0.059 | 0.009 | 2.57E-12 | 0.043 | 0.023 | 6.37E-02 | 86859 | glgc_gwas | intron_variant |
| CAD | HMGCR | rs3846662 | 5 | 74651084 | G | A | 0.477 | 0.065 | 0.003 | 1.00E-200 | 0.028 | 0.015 | 6.20E-02 | 295826 | glgc_exome | non_coding_transcript_exon_variant |
| CAD | HMGCR | rs3846663 | 5 | 74655726 | T | C | 0.395 | 0.066 | 0.003 | 1.00E-200 | 0.034 | 0.014 | 1.76E-02 | 289910 | glgc_exome | intron_variant |
| CAD | HMGCR | rs4704209 | 5 | 74634473 | G | A | 0.054 | 0.015 | 0.008 | 2.61E-02 | -0.013 | 0.033 | 6.89E-01 | 170079 | glgc_gwas | intron_variant |
| CAD | HMGCR | rs5909 | 5 | 74656175 | A | G | 0.102 | 0.062 | 0.009 | 4.93E-13 | 0.050 | 0.023 | 3.00E-02 | 89875 | glgc_gwas | 3_prime_UTR_variant |
| CAD | HMGCR | rs7717396 | 5 | 74654779 | T | A | 0.054 | 0.018 | 0.008 | 1.11E-02 | -0.014 | 0.033 | 6.76E-01 | 170054 | glgc_gwas | intron_variant |
| CAD | APOB | rs10199768 | 2 | 21244000 | T | G | 0.385 | 0.097 | 0.004 | 1.00E-200 | 0.032 | 0.014 | 2.96E-02 | 170875 | glgc_gwas | intron_variant |
| CAD | APOB | rs1042031 | 2 | 21225753 | T | C | 0.172 | -0.045 | 0.004 | 3.04E-37 | 0.020 | 0.018 | 2.71E-01 | 295826 | glgc_exome | stop_gained |
| CAD | APOB | rs1042034 | 2 | 21225281 | T | C | 0.745 | 0.039 | 0.003 | 2.13E-35 | 0.031 | 0.017 | 6.33E-02 | 295826 | glgc_exome | missense_variant |
| CAD | APOB | rs11126598 | 2 | 21240364 | G | A | 0.707 | 0.038 | 0.004 | 4.41E-20 | 0.041 | 0.016 | 1.07E-02 | 172991 | glgc_gwas | intron_variant |
| CAD | APOB | rs11676704 | 2 | 21244358 | T | G | 0.844 | 0.001 | 0.005 | 6.16E-01 | 0.011 | 0.022 | 6.32E-01 | 173023 | glgc_gwas | intron_variant |
| CAD | APOB | rs12691202 | 2 | 21249716 | C | T | 0.949 | 0.097 | 0.011 | 8.22E-19 | 0.064 | 0.038 | 9.80E-02 | 109096 | glgc_gwas | missense_variant |
| CAD | APOB | rs12713844 | 2 | 21238413 | G | C | 0.009 | -0.008 | 0.014 | 5.67E-01 | -0.114 | 0.089 | 2.02E-01 | 290263 | glgc_exome | missense_variant |
| CAD | APOB | rs12713956 | 2 | 21241505 | A | G | 0.875 | 0.072 | 0.006 | 1.32E-28 | 0.023 | 0.025 | 3.53E-01 | 173010 | glgc_gwas | intron_variant |
| CAD | APOB | rs12714264 | 2 | 21265518 | A | T | 0.832 | 0.127 | 0.005 | 1.00E-200 | 0.035 | 0.022 | 1.06E-01 | 172866 | glgc_gwas | intron_variant |
| CAD | APOB | rs12720791 | 2 | 21244617 | A | G | 0.001 | 0.076 | 0.021 | 3.66E-04 | 0.066 | 0.059 | 2.62E-01 | 126766 | glgc_gwas | intron_variant |
| CAD | APOB | rs12720796 | 2 | 21261998 | C | A | 0.022 | 0.091 | 0.014 | 1.68E-10 | 0.032 | 0.076 | 6.72E-01 | 153714 | glgc_gwas | intron_variant |
| CAD | APOB | rs12720828 | 2 | 21241744 | C | T | 0.803 | 0.058 | 0.005 | 3.33E-32 | -0.017 | 0.019 | 3.65E-01 | 170560 | glgc_gwas | intron_variant |
| CAD | APOB | rs12720842 | 2 | 21257927 | C | T | 0.021 | 0.099 | 0.012 | 1.88E-15 | -0.040 | 0.051 | 4.34E-01 | 161300 | glgc_gwas | intron_variant |
| CAD | APOB | rs1367117 | 2 | 21263900 | A | G | 0.285 | 0.105 | 0.003 | 1.00E-200 | 0.035 | 0.016 | 2.30E-02 | 295826 | glgc_exome | missense_variant |
| CAD | APOB | rs1469513 | 2 | 21259562 | C | T | 0.381 | 0.096 | 0.004 | 1.00E-200 | 0.031 | 0.014 | 2.83E-02 | 170012 | glgc_gwas | intron_variant |
| CAD | APOB | rs1801695 | 2 | 21224853 | C | T | 0.974 | 0.002 | 0.014 | 7.41E-01 | 0.004 | 0.049 | 9.27E-01 | 105182 | glgc_gwas | missense_variant |
| CAD | APOB | rs1801700 | 2 | 21245813 | A | G | 0.042 | 0.018 | 0.008 | 9.56E-02 | 0.086 | 0.032 | 6.88E-03 | 168112 | glgc_gwas | missense_variant |
| CAD | APOB | rs1801701 | 2 | 21228827 | T | C | 0.081 | 0.046 | 0.005 | 4.87E-21 | -0.047 | 0.025 | 5.70E-02 | 293853 | glgc_exome | missense_variant |
| CAD | APOB | rs1801702 | 2 | 21225485 | G | C | 0.028 | -0.091 | 0.008 | 3.66E-30 | 0.070 | 0.069 | 3.08E-01 | 295826 | glgc_exome | missense_variant |
| CAD | APOB | rs2678379 | 2 | 21226560 | G | A | 0.768 | 0.058 | 0.004 | 1.65E-38 | 0.031 | 0.017 | 6.36E-02 | 173015 | glgc_gwas | intron_variant |
| CAD | APOB | rs2854725 | 2 | 21237786 | T | G | 0.901 | 0.064 | 0.007 | 4.99E-19 | 0.050 | 0.028 | 7.45E-02 | 172961 | glgc_gwas | intron_variant |
| CAD | APOB | rs3749054 | 2 | 21237238 | A | T | 0.793 | 0.052 | 0.005 | 2.97E-27 | -0.022 | 0.018 | 2.24E-01 | 164997 | glgc_gwas | intron_variant |
| CAD | APOB | rs3791980 | 2 | 21245329 | T | G | 0.703 | 0.043 | 0.004 | 9.74E-21 | 0.036 | 0.016 | 2.31E-02 | 144612 | glgc_gwas | intron_variant |
| CAD | APOB | rs3791981 | 2 | 21245367 | A | G | 0.880 | 0.094 | 0.007 | 1.00E-200 | 0.027 | 0.027 | 3.21E-01 | 161484 | glgc_gwas | intron_variant |
| CAD | APOB | rs512535 | 2 | 21267782 | T | C | 0.484 | 0.031 | 0.004 | 2.32E-15 | 0.011 | 0.015 | 4.47E-01 | 172987 | glgc_gwas | upstream_gene_variant |
| CAD | APOB | rs520354 | 2 | 21259612 | A | G | 0.508 | 0.058 | 0.004 | 1.00E-200 | 0.016 | 0.014 | 2.52E-01 | 169950 | glgc_gwas | intron_variant |
| CAD | APOB | rs531819 | 2 | 21263639 | G | T | 0.809 | 0.134 | 0.005 | 1.00E-200 | 0.041 | 0.021 | 4.88E-02 | 173033 | glgc_gwas | intron_variant |
| CAD | APOB | rs533617 | 2 | 21233972 | C | T | 0.039 | -0.127 | 0.007 | 1.00E-200 | -0.096 | 0.045 | 3.38E-02 | 291354 | glgc_exome | missense_variant |
| CAD | APOB | rs550619 | 2 | 21260601 | A | G | 0.888 | 0.087 | 0.007 | 8.69E-37 | 0.037 | 0.027 | 1.75E-01 | 160112 | glgc_gwas | intron_variant |
| CAD | APOB | rs570877 | 2 | 21251040 | G | T | 0.888 | 0.091 | 0.007 | 1.00E-200 | 0.036 | 0.027 | 1.84E-01 | 172986 | glgc_gwas | intron_variant |
| CAD | APOB | rs579826 | 2 | 21261507 | C | T | 0.888 | 0.097 | 0.009 | 7.04E-23 | 0.036 | 0.027 | 1.90E-01 | 89888 | glgc_gwas | intron_variant |
| CAD | APOB | rs6413458 | 2 | 21231592 | G | A | 0.976 | 0.094 | 0.014 | 2.19E-10 | -0.070 | 0.069 | 3.06E-01 | 159056 | glgc_gwas | synonymous_variant |
| CAD | APOB | rs673548 | 2 | 21237544 | A | G | 0.266 | -0.037 | 0.003 | 1.89E-27 | -0.032 | 0.017 | 5.80E-02 | 249281 | glgc_exome | intron_variant |
| CAD | APOB | rs676210 | 2 | 21231524 | A | G | 0.253 | -0.039 | 0.003 | 5.82E-35 | -0.030 | 0.017 | 7.68E-02 | 295826 | glgc_exome | missense_variant |
| CAD | APOB | rs679899 | 2 | 21250914 | A | G | 0.478 | -0.045 | 0.003 | 1.00E-200 | -0.020 | 0.016 | 2.08E-01 | 295826 | glgc_exome | missense_variant |
| CAD | NPC1L1 | rs10264715 | 7 | 44555406 | A | G | 0.199 | 0.022 | 0.004 | 1.65E-06 | 0.027 | 0.023 | 2.31E-01 | 173026 | glgc_gwas | synonymous_variant |
| CAD | NPC1L1 | rs11763759 | 7 | 44570067 | T | C | 0.715 | 0.038 | 0.007 | 3.81E-07 | 0.041 | 0.025 | 9.66E-02 | 86806 | glgc_gwas | intron_variant |
| CAD | NPC1L1 | rs11767718 | 7 | 44553492 | T | C | 0.170 | 0.019 | 0.005 | 9.03E-05 | 0.031 | 0.017 | 6.95E-02 | 173010 | glgc_gwas | intron_variant |
| CAD | NPC1L1 | rs17655652 | 7 | 44580991 | T | C | 0.711 | 0.028 | 0.004 | 2.18E-10 | 0.047 | 0.024 | 4.71E-02 | 162152 | glgc_gwas | upstream_gene_variant |
| CAD | NPC1L1 | rs2072183 | 7 | 44579180 | C | G | 0.237 | 0.039 | 0.005 | 7.12E-16 | -0.041 | 0.061 | 5.06E-01 | 169790 | glgc_gwas | synonymous_variant |
| CAD | NPC1L1 | rs217406 | 7 | 44573761 | G | C | 0.164 | 0.039 | 0.005 | 4.09E-14 | 0.045 | 0.027 | 9.80E-02 | 169605 | glgc_gwas | intron_variant |
| CAD | NPC1L1 | rs217415 | 7 | 44565464 | A | G | 0.195 | 0.030 | 0.007 | 3.46E-05 | 0.010 | 0.022 | 6.65E-01 | 89888 | glgc_gwas | intron_variant |
| CAD | NPC1L1 | rs217419 | 7 | 44563555 | T | C | 0.191 | 0.028 | 0.007 | 5.40E-05 | 0.012 | 0.021 | 5.56E-01 | 89888 | glgc_gwas | intron_variant |
| CAD | NPC1L1 | rs217420 | 7 | 44561901 | C | A | 0.206 | 0.022 | 0.004 | 3.24E-06 | 0.028 | 0.019 | 1.38E-01 | 160051 | glgc_gwas | intron_variant |
| CAD | NPC1L1 | rs217421 | 7 | 44561600 | G | A | 0.211 | 0.021 | 0.004 | 3.35E-06 | 0.023 | 0.018 | 2.08E-01 | 172272 | glgc_gwas | intron_variant |
| CAD | NPC1L1 | rs217426 | 7 | 44558859 | A | C | 0.974 | 0.005 | 0.011 | 6.64E-01 | -0.017 | 0.044 | 7.01E-01 | 164788 | glgc_gwas | intron_variant |
| CAD | NPC1L1 | rs217429 | 7 | 44555199 | C | A | 0.212 | 0.023 | 0.006 | 2.06E-04 | 0.021 | 0.021 | 3.26E-01 | 89882 | glgc_gwas | intron_variant |
| CAD | NPC1L1 | rs217430 | 7 | 44555081 | G | A | 0.212 | 0.020 | 0.004 | 3.61E-06 | 0.029 | 0.021 | 1.74E-01 | 172999 | glgc_gwas | intron_variant |
| CAD | NPC1L1 | rs217432 | 7 | 44553826 | G | A | 0.195 | 0.022 | 0.006 | 5.84E-04 | 0.026 | 0.022 | 2.47E-01 | 89888 | glgc_gwas | intron_variant |
| CAD | NPC1L1 | rs217433 | 7 | 44553496 | C | T | 0.170 | 0.020 | 0.005 | 7.47E-05 | 0.037 | 0.018 | 4.29E-02 | 173018 | glgc_gwas | intron_variant |
| CAD | NPC1L1 | rs217434 | 7 | 44553238 | G | A | 0.170 | 0.017 | 0.005 | 2.60E-03 | 0.036 | 0.024 | 1.36E-01 | 145288 | glgc_gwas | synonymous_variant |
| CAD | NPC1L1 | rs3187907 | 7 | 44552209 | C | T | 0.170 | 0.018 | 0.004 | 1.09E-04 | 0.030 | 0.017 | 6.76E-02 | 173026 | glgc_gwas | 3_prime_UTR_variant |
| CAD | PCSK9 | rs10888896 | 1 | 55509213 | C | G | 0.720 | 0.043 | 0.005 | 2.14E-14 | -0.044 | 0.059 | 4.52E-01 | 163741 | glgc_gwas | intron_variant |
| CAD | PCSK9 | rs10888897 | 1 | 55513061 | C | T | 0.605 | 0.051 | 0.004 | 8.43E-31 | 0.024 | 0.020 | 2.45E-01 | 165232 | glgc_gwas | intron_variant |
| CAD | PCSK9 | rs11206514 | 1 | 55516004 | A | C | 0.611 | 0.051 | 0.004 | 9.95E-33 | 0.015 | 0.029 | 6.15E-01 | 172996 | glgc_gwas | intron_variant |
| CAD | PCSK9 | rs11206516 | 1 | 55524971 | C | T | 0.016 | -0.003 | 0.043 | 9.42E-01 | -0.086 | 0.099 | 3.85E-01 | 17777 | magnetic | intron_variant |
| CAD | PCSK9 | rs11583680 | 1 | 55505668 | T | C | 0.130 | -0.023 | 0.005 | 1.07E-06 | -0.023 | 0.032 | 4.79E-01 | 202725 | glgc_exome | missense_variant |
| CAD | PCSK9 | rs12067569 | 1 | 55528629 | A | G | 0.034 | 0.089 | 0.010 | 1.97E-17 | 0.041 | 0.034 | 2.35E-01 | 164264 | glgc_gwas | intron_variant |
| CAD | PCSK9 | rs17111555 | 1 | 55529602 | T | C | 0.016 | -0.002 | 0.042 | 9.56E-01 | -0.086 | 0.098 | 3.80E-01 | 17773 | magnetic | 3_prime_UTR_variant |
| CAD | PCSK9 | rs17111557 | 1 | 55529871 | T | C | 0.016 | -0.002 | 0.042 | 9.72E-01 | -0.086 | 0.098 | 3.80E-01 | 17776 | magnetic | 3_prime_UTR_variant |
| CAD | PCSK9 | rs2479409 | 1 | 55504650 | A | G | 0.657 | -0.047 | 0.003 | 1.00E-200 | -0.042 | 0.016 | 1.11E-02 | 295826 | glgc_exome | upstream_gene_variant |
| CAD | PCSK9 | rs2479411 | 1 | 55509900 | T | C | 0.055 | 0.003 | 0.009 | 4.45E-01 | -0.001 | 0.036 | 9.71E-01 | 167215 | glgc_gwas | intron_variant |
| CAD | PCSK9 | rs2495478 | 1 | 55512995 | A | G | 0.055 | 0.003 | 0.009 | 4.04E-01 | 0.000 | 0.036 | 9.92E-01 | 172338 | glgc_gwas | intron_variant |
| CAD | PCSK9 | rs2495480 | 1 | 55509355 | C | T | 0.055 | 0.003 | 0.009 | 5.03E-01 | 0.009 | 0.037 | 8.07E-01 | 170545 | glgc_gwas | intron_variant |
| CAD | PCSK9 | rs2495481 | 1 | 55508186 | T | A | 0.055 | 0.008 | 0.009 | 2.39E-01 | 0.000 | 0.036 | 9.97E-01 | 172296 | glgc_gwas | intron_variant |
| CAD | PCSK9 | rs4927193 | 1 | 55509872 | T | C | 0.869 | 0.035 | 0.006 | 4.27E-11 | 0.021 | 0.029 | 4.68E-01 | 173009 | glgc_gwas | intron_variant |
| CAD | PCSK9 | rs499718 | 1 | 55512549 | C | T | 0.826 | 0.036 | 0.007 | 1.13E-06 | 0.013 | 0.026 | 6.29E-01 | 89888 | glgc_gwas | intron_variant |
| CAD | PCSK9 | rs505151 | 1 | 55529187 | A | G | 0.949 | -0.090 | 0.006 | 1.00E-200 | -0.045 | 0.034 | 1.89E-01 | 290057 | glgc_exome | missense_variant |
| CAD | PCSK9 | rs516499 | 1 | 55526685 | A | G | 0.815 | 0.054 | 0.007 | 5.61E-15 | 0.019 | 0.018 | 2.90E-01 | 89888 | glgc_gwas | intron_variant |
| CAD | PCSK9 | rs533375 | 1 | 55523361 | G | A | 0.815 | 0.063 | 0.005 | 1.52E-33 | 0.013 | 0.020 | 4.94E-01 | 167764 | glgc_gwas | intron_variant |
| CAD | PCSK9 | rs535471 | 1 | 55514182 | C | T | 0.831 | 0.033 | 0.008 | 2.78E-05 | 0.007 | 0.027 | 8.03E-01 | 89888 | glgc_gwas | intron_variant |
| CAD | PCSK9 | rs540796 | 1 | 55524197 | G | A | 0.815 | 0.063 | 0.005 | 4.62E-35 | 0.017 | 0.019 | 3.57E-01 | 167749 | glgc_gwas | missense_variant |
| CAD | PCSK9 | rs557435 | 1 | 55520864 | G | A | 0.792 | 0.062 | 0.007 | 6.27E-20 | 0.020 | 0.025 | 4.31E-01 | 106382 | glgc_gwas | intron_variant |
| CAD | PCSK9 | rs562556 | 1 | 55524237 | A | G | 0.835 | 0.043 | 0.004 | 1.23E-32 | 0.019 | 0.021 | 3.64E-01 | 290057 | glgc_exome | missense_variant |
| CAD | PCSK9 | rs584626 | 1 | 55523984 | T | C | 0.815 | 0.062 | 0.005 | 6.84E-35 | 0.013 | 0.019 | 4.99E-01 | 172995 | glgc_gwas | intron_variant |
| CAD | PCSK9 | rs585131 | 1 | 55524116 | T | C | 0.815 | 0.064 | 0.005 | 2.70E-35 | 0.017 | 0.019 | 3.59E-01 | 167769 | glgc_gwas | intron_variant |
| CAD | PCSK9 | rs602705 | 1 | 55525726 | G | T | 0.815 | 0.051 | 0.007 | 5.06E-13 | 0.018 | 0.019 | 3.52E-01 | 89888 | glgc_gwas | intron_variant |
| CAD | PCSK9 | rs603247 | 1 | 55525868 | T | C | 0.815 | 0.052 | 0.007 | 9.47E-14 | 0.018 | 0.019 | 3.52E-01 | 89888 | glgc_gwas | intron_variant |
| CAD | PCSK9 | rs615563 | 1 | 55526296 | G | A | 0.694 | 0.038 | 0.006 | 8.63E-11 | 0.018 | 0.016 | 2.50E-01 | 89888 | glgc_gwas | intron_variant |
| CAD | PCSK9 | rs630431 | 1 | 55527323 | A | G | 0.691 | 0.035 | 0.004 | 7.73E-17 | 0.018 | 0.016 | 2.42E-01 | 166988 | glgc_gwas | intron_variant |
| CAD | PCSK9 | rs643257 | 1 | 55527918 | T | C | 0.815 | 0.053 | 0.007 | 4.40E-14 | 0.018 | 0.019 | 3.53E-01 | 89138 | glgc_gwas | intron_variant |
| CAD | PCSK9 | rs662145 | 1 | 55529828 | T | C | 0.753 | 0.005 | 0.005 | 2.49E-01 | -0.006 | 0.017 | 7.36E-01 | 110022 | glgc_gwas | 3_prime_UTR_variant |
| CAD | PCSK9 | rs7552841 | 1 | 55518752 | T | C | 0.365 | 0.037 | 0.004 | 5.40E-15 | 0.006 | 0.021 | 7.92E-01 | 140234 | glgc_gwas | intron_variant |
| CAD | PCSK9 | rs9326034 | 1 | 55530664 | A | G | 0.016 | -0.002 | 0.042 | 9.72E-01 | -0.086 | 0.098 | 3.80E-01 | 17777 | magnetic | downstream_gene_variant |
| T2D | APOB | rs10199768 | 2 | 21244000 | T | G | 0.385 | 0.097 | 0.004 | 1.00E-200 | 0.010 | 0.020 | 6.90E-01 | 170875 | glgc_gwas | intron_variant |
| T2D | APOB | rs1042031 | 2 | 21225753 | T | C | 0.172 | -0.045 | 0.004 | 3.04E-37 | 0.000 | 0.023 | 9.40E-01 | 295826 | glgc_exome | stop_gained |
| T2D | APOB | rs11676704 | 2 | 21244358 | T | G | 0.844 | 0.001 | 0.005 | 6.16E-01 | 0.030 | 0.027 | 2.30E-01 | 173023 | glgc_gwas | intron_variant |
| T2D | APOB | rs12720796 | 2 | 21261998 | C | A | 0.022 | 0.091 | 0.014 | 1.68E-10 | 0.000 | 0.096 | 9.80E-01 | 153714 | glgc_gwas | intron_variant |
| T2D | APOB | rs12720842 | 2 | 21257927 | C | T | 0.021 | 0.099 | 0.012 | 1.88E-15 | -0.020 | 0.065 | 7.40E-01 | 161300 | glgc_gwas | intron_variant |
| T2D | APOB | rs1367117 | 2 | 21263900 | A | G | 0.285 | 0.105 | 0.003 | 1.00E-200 | 0.020 | 0.020 | 3.90E-01 | 295826 | glgc_exome | missense_variant |
| T2D | APOB | rs1801700 | 2 | 21245813 | A | G | 0.042 | 0.018 | 0.008 | 9.56E-02 | -0.010 | 0.043 | 8.20E-01 | 168112 | glgc_gwas | missense_variant |
| T2D | APOB | rs1801701 | 2 | 21228827 | T | C | 0.081 | 0.046 | 0.005 | 4.87E-21 | 0.039 | 0.032 | 2.20E-01 | 293853 | glgc_exome | missense_variant |
| T2D | APOB | rs2678379 | 2 | 21226560 | G | A | 0.768 | 0.058 | 0.004 | 1.65E-38 | 0.000 | 0.023 | 9.10E-01 | 173015 | glgc_gwas | intron_variant |
| T2D | APOB | rs2854725 | 2 | 21237786 | T | G | 0.901 | 0.064 | 0.007 | 4.99E-19 | 0.000 | 0.038 | 9.50E-01 | 172961 | glgc_gwas | intron_variant |
| T2D | APOB | rs3791981 | 2 | 21245367 | A | G | 0.880 | 0.094 | 0.007 | 1.00E-200 | -0.020 | 0.032 | 5.40E-01 | 161484 | glgc_gwas | intron_variant |
| T2D | APOB | rs512535 | 2 | 21267782 | T | C | 0.484 | 0.031 | 0.004 | 2.32E-15 | 0.020 | 0.020 | 2.90E-01 | 172987 | glgc_gwas | upstream_gene_variant |
| T2D | APOB | rs531819 | 2 | 21263639 | G | T | 0.809 | 0.134 | 0.005 | 1.00E-200 | -0.010 | 0.028 | 6.90E-01 | 173033 | glgc_gwas | intron_variant |
| T2D | APOB | rs533617 | 2 | 21233972 | C | T | 0.039 | -0.127 | 0.007 | 1.00E-200 | -0.077 | 0.064 | 2.50E-01 | 291354 | glgc_exome | missense_variant |
| T2D | APOB | rs6413458 | 2 | 21231592 | G | A | 0.976 | 0.094 | 0.014 | 2.19E-10 | -0.039 | 0.073 | 6.00E-01 | 159056 | glgc_gwas | synonymous_variant |
| T2D | APOB | rs679899 | 2 | 21250914 | A | G | 0.478 | -0.045 | 0.003 | 1.00E-200 | 0.020 | 0.040 | 6.40E-01 | 295826 | glgc_exome | missense_variant |
| T2D | HMGCR | rs10045497 | 5 | 74636484 | A | C | 0.408 | 0.077 | 0.005 | 1.00E-200 | -0.020 | 0.020 | 2.60E-01 | 89888 | glgc_gwas | intron_variant |
| T2D | HMGCR | rs10474435 | 5 | 74657280 | C | T | 0.009 | 0.054 | 0.015 | 2.36E-03 | -0.157 | 0.078 | 3.90E-02 | 150792 | glgc_gwas | 3_prime_UTR_variant |
| T2D | HMGCR | rs10515198 | 5 | 74641560 | A | G | 0.103 | 0.060 | 0.006 | 5.99E-22 | 0.010 | 0.033 | 7.00E-01 | 173012 | glgc_gwas | intron_variant |
| T2D | HMGCR | rs2303151 | 5 | 74655451 | T | C | 0.054 | 0.015 | 0.008 | 2.20E-02 | -0.068 | 0.040 | 8.60E-02 | 170080 | glgc_gwas | non_coding_transcript_exon_variant |
| T2D | HMGCR | rs2303152 | 5 | 74641707 | A | G | 0.120 | 0.042 | 0.006 | 1.04E-09 | 0.030 | 0.032 | 3.40E-01 | 160116 | glgc_gwas | intron_variant |
| T2D | HMGCR | rs5908 | 5 | 74652199 | G | A | 0.018 | -0.036 | 0.010 | 2.32E-04 | -0.039 | 0.111 | 7.10E-01 | 290263 | glgc_exome | missense_variant |
| T2D | NPC1L1 | rs10264715 | 7 | 44555406 | A | G | 0.199 | 0.022 | 0.004 | 1.65E-06 | 0.000 | 0.023 | 8.70E-01 | 173026 | glgc_gwas | synonymous_variant |
| T2D | NPC1L1 | rs17655652 | 7 | 44580991 | T | C | 0.711 | 0.028 | 0.004 | 2.18E-10 | -0.049 | 0.024 | 4.20E-02 | 162152 | glgc_gwas | upstream_gene_variant |
| T2D | NPC1L1 | rs217426 | 7 | 44558859 | A | C | 0.974 | 0.005 | 0.011 | 6.64E-01 | 0.049 | 0.051 | 3.70E-01 | 164788 | glgc_gwas | intron_variant |
| T2D | NPC1L1 | rs217432 | 7 | 44553826 | G | A | 0.195 | 0.022 | 0.006 | 5.84E-04 | -0.010 | 0.023 | 6.10E-01 | 89888 | glgc_gwas | intron_variant |
| T2D | NPC1L1 | rs4720470 | 7 | 44561884 | C | T | 0.933 | 0.002 | 0.010 | 9.44E-01 | 0.113 | 0.106 | 2.80E-01 | 117734 | glgc_gwas | intron_variant |
| T2D | PCSK9 | rs11206514 | 1 | 55516004 | A | C | 0.611 | 0.051 | 0.004 | 9.95E-33 | -0.010 | 0.035 | 8.00E-01 | 172996 | glgc_gwas | intron_variant |
| T2D | PCSK9 | rs2479409 | 1 | 55504650 | A | G | 0.657 | -0.047 | 0.003 | 1.00E-200 | 0.020 | 0.020 | 3.00E-01 | 295826 | glgc_exome | upstream_gene_variant |
| T2D | PCSK9 | rs2495478 | 1 | 55512995 | A | G | 0.055 | 0.003 | 0.009 | 4.04E-01 | -0.020 | 0.050 | 7.20E-01 | 172338 | glgc_gwas | intron_variant |
| T2D | PCSK9 | rs4927193 | 1 | 55509872 | T | C | 0.869 | 0.035 | 0.006 | 4.27E-11 | 0.049 | 0.034 | 2.10E-01 | 173009 | glgc_gwas | intron_variant |
| T2D | PCSK9 | rs499718 | 1 | 55512549 | C | T | 0.826 | 0.036 | 0.007 | 1.13E-06 | 0.039 | 0.029 | 2.20E-01 | 89888 | glgc_gwas | intron_variant |
| T2D | PCSK9 | rs505151 | 1 | 55529187 | A | G | 0.949 | -0.090 | 0.006 | 1.00E-200 | 0.000 | 0.043 | 9.50E-01 | 290057 | glgc_exome | missense_variant |
| T2D | PCSK9 | rs557435 | 1 | 55520864 | G | A | 0.792 | 0.062 | 0.007 | 6.27E-20 | -0.030 | 0.027 | 3.30E-01 | 106382 | glgc_gwas | intron_variant |
| T2D | PCSK9 | rs572512 | 1 | 55517344 | T | C | 0.346 | 0.048 | 0.005 | 5.31E-26 | -0.030 | 0.054 | 5.80E-01 | 150564 | glgc_gwas | non_coding_transcript_exon_variant |
| T2D | PCSK9 | rs585131 | 1 | 55524116 | T | C | 0.815 | 0.064 | 0.005 | 2.70E-35 | -0.039 | 0.025 | 1.00E-01 | 167769 | glgc_gwas | intron_variant |
| T2D | PCSK9 | rs630431 | 1 | 55527323 | A | G | 0.691 | 0.035 | 0.004 | 7.73E-17 | -0.030 | 0.022 | 9.50E-02 | 166988 | glgc_gwas | intron_variant |
| T2D | PCSK9 | rs7552841 | 1 | 55518752 | T | C | 0.365 | 0.037 | 0.004 | 5.40E-15 | 0.000 | 0.023 | 9.10E-01 | 140234 | glgc_gwas | intron_variant |

*Abbreviations: CAD - Coronary Artery Disease; Chr – chromosome; EAF - effect allele frequency; glgc_exome - Global Lipid Genetics Consortium exome-wide study; glgc_gwas - Global Lipid Genetics Consortium genome-wide association study; SNP - single nucleotide polymorphism; Stroke - ischemic stroke; T2D - type-2 diabetes mellitus*

Foot notes:

1) SNP positions are from human reference genome build GRChr37

2) P values for association with LDL-C were recorded with a lower limit of 1.00E-200

3) Functional annotations for SNPs were derived from Ensembl Variant Effect Predictor

# Supplementary table 5: information on genome-wide variants used to assess the effects of lowering circulating PCSK9 on AD and CAD

| **Outcome** | **SNP** | **Chr** | **Position** | **Effect allele** | **Alt allele** | **EAF exposure** | **Beta exposure** | **SE exposure** | **P exposure** | **Beta outcome** | **SE outcome** | **P outcome** | **Sample size exposure** | **Functional consequence** |
| --- | --- | --- | --- | --- | --- | --- | --- | --- | --- | --- | --- | --- | --- | --- |
| IGAP_2019 | rs11591147 | 1 | 55505647 | T | G | 0.012 | -0.374 | 0.046 | 5.91E-16 | 0.030 | 0.078 | 6.95E-01 | 2022 | missense_variant |
| IGAP_2019 | rs45448095 | 1 | 55505447 | T | C | 0.133 | -0.082 | 0.015 | 3.19E-08 | 0.007 | 0.021 | 7.30E-01 | 2022 | 5_prime_UTR_variant |
| IGAP_2019 | rs6957201 | 7 | 5475473 | C | T | 0.971 | -0.167 | 0.035 | 1.70E-06 | 0.020 | 0.038 | 6.03E-01 | 2022 | intron_variant |
| PGC | rs11591147 | 1 | 55505647 | T | G | 0.012 | -0.374 | 0.046 | 5.91E-16 | 0.198 | 0.182 | 2.76E-01 | 2022 | missense_variant |
| PGC | rs45448095 | 1 | 55505447 | T | C | 0.133 | -0.082 | 0.015 | 3.19E-08 | 0.045 | 0.055 | 4.13E-01 | 2022 | 5_prime_UTR_variant |
| PGC | rs6957201 | 7 | 5475473 | C | T | 0.971 | -0.167 | 0.035 | 1.70E-06 | 0.177 | 0.106 | 9.60E-02 | 2022 | intron_variant |
| CAD | rs2479409 | 1 | 55504650 | A | G | 0.640 | -0.057 | 0.011 | 3.19E-07 | -0.042 | 0.016 | 1.11E-02 | 2022 | upstream_gene_variant |
| CAD | rs45448095 | 1 | 55505447 | T | C | 0.133 | -0.082 | 0.015 | 3.19E-08 | -0.023 | 0.032 | 4.79E-01 | 2022 | 5_prime_UTR_variant |

*Abbreviations: CAD - Coronary Artery Disease; Chr – chromosome; EAF - effect allele frequency; IGAP - International Genomics of Alzheimer's disease project; PGC - Psychiatric Genomics Consortium; SNP - single nucleotide polymorphism*

Foot notes:

1) SNP positions are from human reference genome build GRChr37

2) Functional annotations for SNPs were derived from Ensembl Variant Effect Predictor

# Supplementary table 6: Alternate MR methods for examining gene region variants in relation to AD risk, using two LD-clumping strategies instead of principal component methodology

| **Clumping strategy** | **Study** | **Gene** | **SNPs** | **Method** | **OR** | **CI** |
| --- | --- | --- | --- | --- | --- | --- |
| Conservative (r2<0.01) | IGAP_2019 | HMGCR | 2 | IVW | 0.84 | (0.54, 1.31) |
|  |  | PCSK9 | 4 | IVW | 1.17 | (0.67, 2.04) |
|  |  |  |  | MR Egger | 0.37 | (0.08, 1.65) |
|  |  |  |  | Weighted median | 0.97 | (0.69, 1.38) |
|  |  | APOB | 5 | IVW | 1.22 | (0.92, 1.60) |
|  |  |  |  | MR Egger | 1.52 | (0.64, 3.64) |
|  |  |  |  | Weighted median | 1.29 | (0.92, 1.80) |
|  |  | NPC1L1 | 1 | Wald ratio | 1.90 | (0.88, 4.08) |
|  | PGC | HMGCR | 2 | IVW | 0.59 | (0.12, 3.01) |
|  |  | PCSK9 | 4 | IVW | 1.90 | (0.99, 3.64) |
|  |  |  | 4 | MR Egger | 0.98 | (0.10, 9.51) |
|  |  |  | 4 | Weighted median | 1.85 | (0.80, 4.26) |
|  |  | APOB | 6 | IVW | 0.88 | (0.44, 1.78) |
|  |  |  | 6 | MR Egger | 2.47 | (0.34, 18.17) |
|  |  |  | 6 | Weighted median | 0.82 | (0.33, 2.04) |
|  |  | NPC1L1 | 1 | Wald ratio | 0.42 | (0.07, 2.65) |
| Liberal (r2<0.2) | IGAP_2019 | HMGCR | 5 | IVW | 0.94 | (0.57, 1.54) |
|  |  |  |  | MR Egger | 0.70 | (0.05, 9.23) |
|  |  |  |  | Weighted median | 0.84 | (0.58, 1.21) |
|  |  | PCSK9 | 13 | IVW | 1.24 | (0.99, 1.56) |
|  |  |  |  | MR Egger | 0.95 | (0.63, 1.42) |
|  |  |  |  | Weighted median | 1.06 | (0.86, 1.32) |
|  |  | APOB | 17 | IVW | 1.03 | (0.89, 1.19) |
|  |  |  |  | MR Egger | 1.21 | (0.85, 1.73) |
|  |  |  |  | Weighted median | 1.05 | (0.87, 1.26) |
|  |  | NPC1L1 | 2 | IVW | 1.52 | (0.88, 2.63) |
|  | PGC | HMGCR | 5 | IVW | 0.90 | (0.28, 2.89) |
|  |  |  | 5 | MR Egger | 0.12 | (0.00, 30.67) |
|  |  |  | 5 | Weighted median | 0.74 | (0.23, 2.42) |
|  |  | PCSK9 | 12 | IVW | 1.79 | (1.22, 2.63) |
|  |  |  | 12 | MR Egger | 1.54 | (0.74, 3.22) |
|  |  |  | 12 | Weighted median | 1.76 | (1.06, 2.91) |
|  |  | APOB | 17 | IVW | 0.95 | (0.67, 1.35) |
|  |  |  | 17 | MR Egger | 1.09 | (0.46, 2.58) |
|  |  |  | 17 | Weighted median | 0.93 | (0.59, 1.45) |
|  |  | NPC1L1 | 2 | IVW | 0.44 | (0.11, 1.74) |

*Abbreviations: IGAP - International Genomics of Alzheimer's disease project; IVW - inverse variance weighted; PGC - Psychiatric Genomics Consortium; SNP - single nucleotide polymorphism*

All risk estimates are per standard deviation lower LDL-C
